# Towards smart scanning probe lithography: a framework accelerating nano-fabrication process with in-situ characterization via machine learning

**DOI:** 10.1038/s41378-023-00587-z

**Published:** 2023-10-10

**Authors:** Yijie Liu, Xuexuan Li, Ben Pei, Lin Ge, Zhuo Xiong, Zhen Zhang

**Affiliations:** 1 State Key Laboratory of Tribology in Advanced Equipment, Department of Mechanical Engineering, Tsinghua University, Beijing, 100084 China; 2 Beijing Key Laboratory of Precision/Ultra-precision Manufacturing Equipments and Control, Tsinghua University, Beijing, 100084 China; 3 Biomanufacturing Center, Department of Mechanical Engineering, Tsinghua University, Beijing, 100084 China; 4 Biomanufacturing and Rapid Forming Technology Key Laboratory of Beijing, Beijing, 100084 China; 5 ‘Biomanufacturing and Engineering Living Systems’ Innovation International Talents Base (111 Base), Beijing, 100084 China; 6 NT-MDT Spectrum Instruments China office, Beijing, 100053 China

**Keywords:** Nanoscience and technology, Engineering

## Abstract

Scanning probe lithography (SPL) is a promising technology to fabricate high-resolution, customized and cost-effective features at the nanoscale. However, the quality of nano-fabrication, particularly the critical dimension, is significantly influenced by various SPL fabrication techniques and their corresponding process parameters. Meanwhile, the identification and measurement of nano-fabrication features are very time-consuming and subjective. To tackle these challenges, we propose a novel framework for process parameter optimization and feature segmentation of SPL via machine learning (ML). Different from traditional SPL techniques that rely on manual labeling-based experimental methods, the proposed framework intelligently extracts reliable and global information for statistical analysis to fine-tune and optimize process parameters. Based on the proposed framework, we realized the processing of smaller critical dimensions through the optimization of process parameters, and performed direct-write nano-lithography on a large scale. Furthermore, data-driven feature extraction and analysis could potentially provide guidance for other characterization methods and fabrication quality optimization.

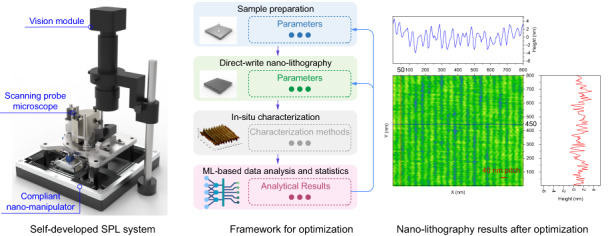

## Introduction

Direct-write nano-lithography is a maskless, serial technique in which beams or probes are scanned through photoresists to create intricate and fine structures at the nanoscale. This technique plays a pivotal role in the fabrication of ultimately scaled devices and photomasks for high volume semiconductors manufacturing, such as deep ultraviolet lithography (DUV) and extreme ultraviolet lithography (EUV)^1^. Currently, the most widely used direct-write nano-lithography methods mainly include electron beam lithogram (EBL)^2^, focused ion beam (FIB)^3^, and scanning probe lithography (SPL)^4^. A practical advantage of EBL and FIB is that they support nano-lithography at a variety of materials, such as polymers^5,6^, alloys^7^, metals and metal-containing ceramics^8^. However, the equipment of EBL is relatively expensive and complex due to electron-compatible optics. Another issue with EBL and FIB are affected by proximity effects, which can negatively impact the accuracy of fabricated nano-structures^9^. Additionally, these techniques require operation in an ultra-high vacuum (UHV) environment. SPL, on the other hand, is a versatile player in the nano-lithography field as it supports various fabrication methods without harsh requirements for materials or environments. Furthermore, SPL is cost-effective, compact, and enables in-situ characterization while maintaining nano-lithography capability. Depending on the interaction mechanism between the probe and the sample surface, SPL can be categorised into close-to-atomic scale SPL^10,11^, oxidation SPL (o-SPL)^12,13^, thermal SPL (t-SPL)^1,14^, thermochemical SPL (tc-SPL)^15,16^, dip-pen SPL (d-SPL)^17,18^, bias-induced SPL (b-SPL)^19,20^ and mechanical SPL (m-SPL)^21–23^.

The fabrication results of the above-mentioned SPL approaches are significantly affected by the conditions and parameters associated with sample preparation and nano-lithography. Ideal sample preparation is an essential prerequisite for producing fine nano-structures in nano-lithography. Notably, the lithography mechanism is directly affected by the sample material and probe type. Additionally, the critical dimensions of features are significantly correlated with the thickness of the polymeric resist on the substrate, while the spin coating speed is the principal factor determining the thickness of polymeric resist. Furthermore, environmental conditions also have the potential to affect the fabrication results. And the existing results indicate a link between critical dimensions of nano-structures and process parameters in nano-lithography. As illustrative cases, we here consider b-SPL and m-SPL. Bias voltage, current and scanning speed have been identified as major contributing factors to the feature size of b-SPL^19,24^. And the fabrication result of nano-lithography with m-SPL can be attributed to the parameters of drive amplitude, tip radius and scanning speed.

The fabrication results of nano-lithography are substantially impacted by various process conditions and parameters. In particular, there may be complex coupling relationships between different parameters. In order to achieve the desired critical dimensions and uniform features, it is essential to establish a comprehensive and unified in-situ characterization and metrology of the nano-lithography results. Although selecting one or more cross-sectional lines for manual measurement of nano-structures is a commonly used method^25–27^, fully characterizing and measuring the entire nano-structures is often difficult and time-consuming. This presents a significant challenge for improving and optimizing process parameters.

Recent advances in artificial intelligence (AI) are shaping the future of nearly every industry. Machine learning (ML) techniques have been developed and applied to manufacturing for modeling^28^, optimization^29,30^, control^31^, monitoring^32^, and prediction^33,34^. In terms of fabrication and characterization of the nanoscale, ML plays a pivotal role in aspect of identification of nanotubes^35^, image super-resolution^36^, nano-structure detection^37^, feature segmentation^38^ and electrostatic characterization^39^. It should be noted that existing studies of ML-based approaches for the nanoscale have mainly focused on improving imaging and analysis. Therefore, there is an urgent need yet a crucial challenge to propose a framework for optimizing process conditions and parameters with reliable and global information for statistical analysis in SPL.

In this paper, we propose a novel ML-based framework for optimizing process conditions and parameters, thereby accelerating nano-fabrication process with in-situ characterization. The proposed framework consists of a semantic segmentation approach to extract global information and features from the in-situ characterization results of SPL. This enables automatic real-time acquisition of global information for statistical analysis. Moreover, we provide reliable, uniform and reasonable statistic metrics for nano-lithography, which is simply employed to improve and optimize process conditions and parameters. In addition, the data-driven feature extraction and analysis could potentially be applied to other characterization methods and optimization of fabrication quality. Different from existing experimental methods that rely on manually labeled and processed data, our framework enables rapid optimization of process conditions and parameters in nano-lithography based on automatically processed global and uniform information from in-situ characterization.

The rest of the paper is organized as follows. The methodology of the proposed framework for nano-fabrication process are described in Section “Methodology”. Section “Results” discusses the experimental results of automatic statistics, analysis, and optimization of process conditions and parameters based on the framework, followed by large-area nano-lithography results. Finally, conclusions are presented in Section “Conclusion”.

## Methodology

The methodology and framework presented in this paper have a universal applicability for the optimization of process parameters and the segmentation of features in nano-fabrication. In addition, we introduce a nano-manipulator-based SPL system that supports direct-write nano-lithography and in-situ characterization at the macroscale, which we use to demonstrate the methodology and framework.

### Compliant nano-manipulator-based SPL system

The scanning probe microscope (SPM) tip-based nano-fabrication shows great advantages in efficiency, compactness and cost. However, the motion range of the piezoelectric tube scanner that drives the scanning probe or sample stage is generally limited to less than 100 μm × 100 μm × 10 μm. One of the key challenges in the development of the next-generation SPL is to enable direct-write nano-lithography and in-situ characterization at the macroscale^40^. To achieve larger area nano-lithography, a macro motion stage needs to be provided to switch the fabrication area of the scanning system. In the conventional “step and scan” processing method, the large-area pattern is divided into multiple sub-patterns and subsequently stitched together to form a large-area pattern. Nevertheless, this approach is accompanied by unavoidable stitching errors at the junctions of different sub-patterns.

**Fig. 1:**
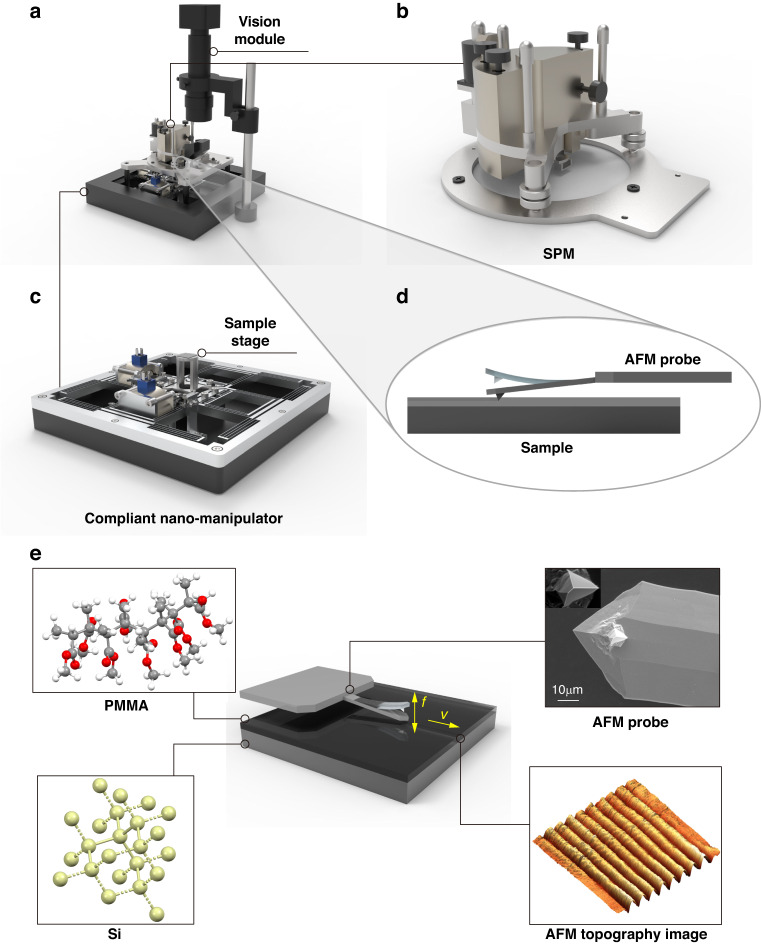
Design and assembly of the proposed compliant nano-manipulator-based SPL system. **a** Design of the SPL system with an SPM, a compliant nano-manipulator and a vision module. **b** SPM. **c** Compliant nano-manipulator. **d** A magnified schematic of the sample and the scanning probe of SPM. **e** Nano-lithography and in-situ characterization process

In order to address the issues of stitching errors and low fabrication efficiency resulting from the “step and scan” processing method described above, a compliant nano-manipulator-based SPL system is proposed. The nano-lithography system, depicted in Fig. 1a, comprises three components, namely an SPM, a compliant nano-manipulator, and a vision module. The SPM, as shown in Fig. 1b, performs nano-lithography and in-situ characterization within the range of tens of microns. The compliant nano-manipulator, demonstrated in Fig. 1c, provides continuous motion with nanometric precision and millimeter-level stroke^41^, due to its friction-free, hysteresis-free, and high linearity characteristics^41,42^ (see Section S1–S4 of the Supplementary Information for the details of the design and the prototype of the compliant nano-manipulator). In the SPL system, the compliant nano-manipulator propels samples to accomplish nano-fabrication in a millimeter range without any stitching. While vision module is employed to observe the scanning probe and sample at the macroscale. During the nano-fabrication process, the probe interacts with the polymeric resist on the surface of the sample, as shown in Fig. 1d.

The combination of compliant nano-manipulator and SPM enables a wide range of motion modes for nano-lithography and inspection purposes. When operating within the motion range of the SPM, only one motion system is required for nano-lithography. However, in instances where the motion range of the SPM is exceeded, the compliant nano-manipulator comes into play by driving the sample in continuous motion to achieve nano-lithography.

Figure 1e illustrates the process of nano-lithography and in-situ characterization. In order to achieve nano-lithography, it is required to prepare samples first. Prior to nano-lithography, sample preparation is necessary, typically involving a substrate and a layer of polymeric resist spin-coated onto the substrate surface. In this study, polished silicon wafers serve as substrates, while poly(methyl methacrylate) (PMMA) is selected as the polymeric resist. The atomic force microscope (AFM) is a common type of SPM utilized for nano-lithography and in-situ characterization. During the nano-lithography mode, the probe interacts with the sample to achieve fabrication, followed by in-situ characterization of the fabrication results in contact or semi-contact mode using the same probe. And topography is a standard method used for the characterization of nano-lithography. It is worth noting that the determination and optimization of process parameters are crucial for large-area and uniform nano-fabrication. Thus, our study is focused on the determination and optimization of process parameters.

### Prototype system, experiments and data preprocessing

**Fig. 2:**
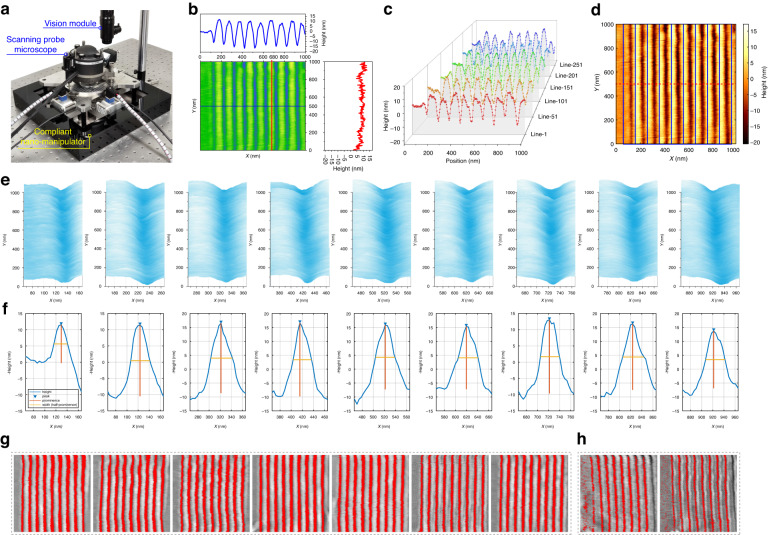
Prototype system and data preprocessing. **a** Prototype system of the compliant nano-manipulator-based SPL system. **b** AFM topography image of the nano-structures. **c** Height distribution of different cross-sectional lines of the nano-structures. **d** Division of different nano-structures regions. **e** 3D topography of individual nano-structures. **f** Analysis of the data in the middle row in the Y direction of the topography for each nano-structure. **g** Grayscale image of AFM topography and automatically identified nano-structures regions (1000 nm × 1000 nm). **h** Failed cases of automatically identified nano-structures regions (1000 nm × 1000 nm)

With the proposed compliant nano-manipulator-based SPL system, we set up a prototype and conduct experiments of nano-lithography and in-situ characterization, as shown in Fig. 2. And the SPL prototype system and its components are shown in Fig. 2a. The SPM used in the system is the commercial AFM head SMENA^TM^ by NT-MDT S.I. Co. (NT-MDT Spectrum Instruments, Moscow, Russia), and the sample is placed on the motion stage of the compliant nano-manipulator. The system is mounted on an optical table to ensure vibration isolation, and can operate under ambient conditions without requiring a vacuum environment.

The substrate used for sample preparation is a standard silicon wafer with dimensions of 20 mm × 20 mm. The substrate has a type N orientation of 111 and a thickness of 625 ± 25 μm, with a resistivity of less than 0.02 Ω ⋅ cm. A thin film of PMMA (molecular weight of 950 k, in solvent chlorobenzene, 1 wt%, AR-P 671.01, Allresist GmbH, Strausberg, Germany) is then spin-coated onto the substrate at a speed of 1000 rpm for 10 s followed by 6,000 rpm for 50 s. The PMMA-coated substrate is then baked on a hotplate at 125 ^∘^C for 30 min for solvent dry-out. The resulting PMMA film thickness is approximately 20 nm.

Dynamic plowing lithography (DPL) is a m-SPL technique for achieving homogeneous nano-structures^43,44^. In DPL, the probe oscillates freely at a high frequency (*f*), while it moves at a constant speed (*v*). During the process, a tapping mode AFM probe with an aluminum reflective coating (Tap190Al-G, BudgetSensors®, Sofia, Bulgaria) is used for nano-lithography. These probes have long cantilevers with conical tips that are 15 μm high at the apex, and a typical curvature radius of approximately 10 nm (see Section S12 of the Supplementary Information for the details of the scanning probe in the SPL system). The nominal spring constant *k* is 48 N/m, and the resonant frequency is 190 kHz. The free amplitude of the probe during the nano-lithography is around 73.4 nm, and the setpoint is expressed as a percentage of the free vibration amplitude, with the setpoint value under free vibration being 10 nA. Different setpoint values are set during the experiment to represent different amplitudes. The experiments are performed in an ambient environment at a room temperature of about 25 ^∘^C and relative humidity of around 50%.

The identical AFM probe is utilized for in-situ characterization of the nano-lithography results. The uninhibited amplitude of the probe throughout the scanning progression is 12 nm. During the process of the sample characterization, the scanning frequency is 1 Hz, whereby 256 lines comprising of 256 pixels each are scanned. The data acquisition is executed employing NOVA_PX software (NT-MDT Spectrum Instruments, Moscow, Russia), and all the data are flattened to the second order. The AFM topography images obtained by scanning in semi-contact mode are analyzed utilizing Image Analysis P9 software (NT-MDT Spectrum Instruments, Moscow, Russia).

Figure 2b shows the AFM topography image of the nano-structures. The nano-structures, consisting of nine nano-grooves with a pitch of 100 nm, are fabricated by DPL. Typically, the data on a transverse line is insufficient to reveal all the information. Therefore, we present the height distribution of five other cross-sectional lines in Fig. 2c that are evenly spaced in Fig. 2b. It is evident that the width and depth of the same groove in nano-lithography vary at different locations. To describe the width of the nano-grooves, the parameter of full width at half maximum (FWHM) is usually adopted. The conventional approach is to select one or multiple cross-sectional lines for manual measurement of the FWHM of the nano-structures. However, it is very error-prone, time-consuming and usually difficult to fully characterize and manually measure the entire nano-structures. As a result, it remains challenging to optimize the process of nano-lithography without an accurate and reasonable metric.

To overcome these challenges, a data preprocessing approach has been proposed for nano-grooves that automatically extracts information regarding their width, depth, and location at each position. It is worth mentioning that the positions of grooves may vary across the scanning area due to different fabrication results. In order to accurately divide the area of each groove, data from half of the positions in the length direction of the groove are chosen for analysis, as shown by the red dash line in Fig. 2d. Using this data, the positions of the vertical grooves are determined in the horizontal direction, and the findpeaks function in the MATLAB® software package is employed to process this data. Since the findpeaks function is used to only detect the peak of the data, the selected data is considered as the opposite number, and the troughs of the grooves are treated as peaks. Consequently, the different regions of the nano-grooves are identified and delineated by the blue boxes in Fig. 2d.

Employing the aforementioned method, we present the 3D topographic representation of nine distinct nano-structures individually in Fig. 2e. And Fig. 2f illustrates the data corresponding to the selected row along with the associated information of the nine grooves. In addition, we obtain groove information, comprising width, depth, and position details in 256 × 9 dimensions, for the entire AFM topography image. Furthermore, we visualize the above information with the AFM topography image. As shown in Fig. 2g, the grayscale representation of AFM topography and the automatically identified FWHM regions of the nano-structures (in red pixels) enable comprehensive assessment of the fabrication results. This data preprocessing method provides a guideline for the nano-lithography results. Nevertheless, in some cases, as depicted in Fig. 2h, this method may not succeed. Furthermore, this method is significantly affected by the noise encountered during the characterization process. The continuous nano-lithography results should be consecutive, but the information obtained by this method may be mutated. Consequently, we employ this method only as a preprocessing result to support our subsequent study. There is thereby an urgent need but it is still a crucial challenge to implement a framework for optimizing process conditions and parameters with reliable global information for statistical analysis in SPL.

### Framework

**Fig. 3:**
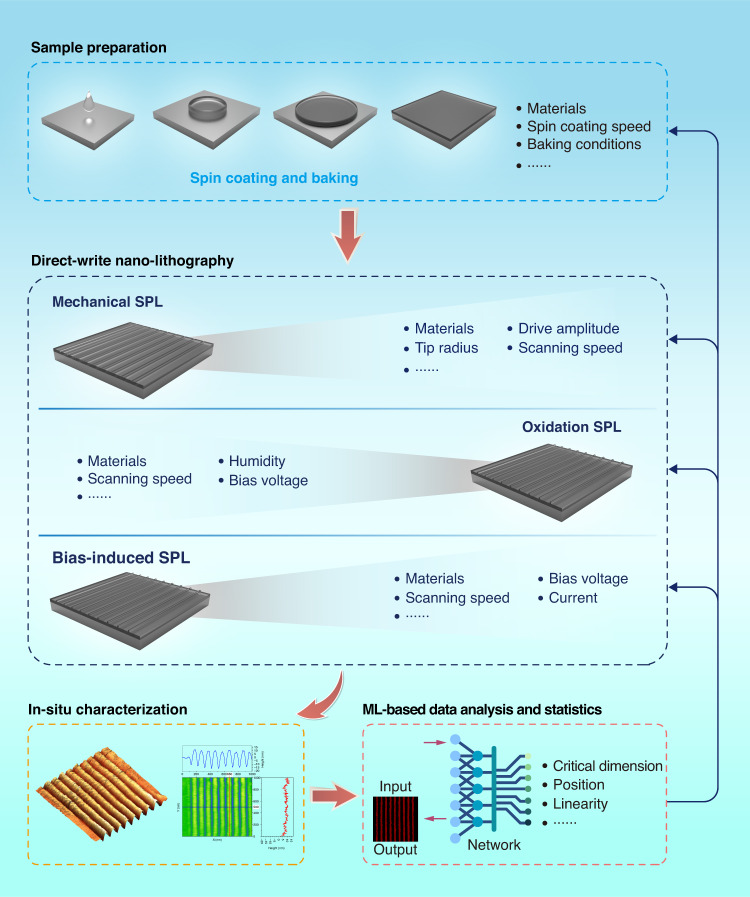
A framework accelerating nano-fabrication process with in situ characterization via ML. The ML-based framework is divided into the following main steps: sample preparation, direct-write nano-lithography, in situ characterization, ML-based data analysis and statistics

It should be pointed out that a comprehensive and uniform evaluation of nano-lithography results is a prerequisite for the optimization of process conditions and parameters, while also expediting the process of parameter optimization and iteration. In this regard, we propose an ML-based full-flow framework for nano-lithography, as shown in Fig. 3.

The proposed general framework encompasses four parts, including sample preparation, direct-write nano-lithography, in-situ characterization, and ML-based data analysis and statistics, as illustrated in Fig. 3. Regarding sample preparation, it is worth noting that the critical dimension of features is closely related to the thickness of the polymeric resist on the substrate, which is primarily determined by the spin coating speed. Additionally, other parameters, such as baking conditions, may also impact the nano-lithography results. In terms of direct-write nano-lithography, we provide examples of m-SPL, o-SPL, and b-SPL in the above framework. Although their processing principles differ considerably, parameters such as scanning speed, drive amplitude, tip radius, bias voltage, and current have a direct influence on the critical dimension of the nano-lithography results. After sample preparation and direct-write nano-lithography, the samples are characterized with the same probes. Unlike conventional nano-fabrication that employs one or more local regions to represent the conditions and parameters, the proposed framework enables the acquisition of global information through real-time ML-based data processing and analysis. This feature allows the framework to effectively guide the optimization of conditions and parameters throughout the nano-lithography process (see Section S6 of the Supplementary Information for the details of the comparison of traditional SPL process parameter optimization method and the proposed one).

**Fig. 4:**
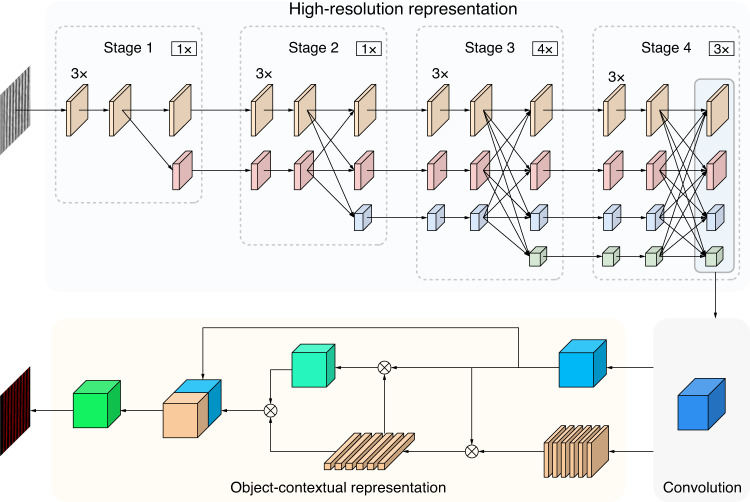
HR-C-OCR semantic segmentation network for processing and analysis of nano-lithography results. The semantic segmentation network is divided into the following main parts: high-resolution representation, convolution and object-contextual representation

In the ML-based framework for nano-lithography, the ML approach is a semantic segmentation network, as shown in Fig. 4. This is a supervised, end-to-end and pixel-by-pixel prediction method for AFM characterization results. Based on the trained model of the network, the nano-structures are rapidly segmented according to the input in-situ characterization results of nano-lithography, and then the resulting statistics and analysis are realized.

The aforementioned semantic segmentation network is a pipeline consisting of encoding and decoding components, encompassing input images, a backbone, decoding heads, segmentation maps, and loss functions. In this case, the input images are grayscale AFM topography images of nano-structures, with a dimension of 256 × 256 pixels. The backbone is an approach based on high-resolution representation (HR)^45^. Previous semantic segmentation methods have mostly obtained semantic information by downsampling, then upsampling to restore high-resolution position information. This, however, leads to a significant loss of valid information during upsampling and downsampling. To overcome this problem and maintain high resolution, feature maps of different resolutions are connected in parallel, and fusion between them is added. In this approach, multi-level features are extracted, and features at different levels are interacted, which can effectively improve the ability of feature extraction. The segmentation map is recovered from the features extracted by the backbone with the aid of cascade decode head based on convolution (C)^46^ and object-contextual representation (OCR)^47^. Although the feature maps can be recovered using only convolution, the OCR decoding head effectively utilizes contextual information to further enhance the accuracy and precision of segmentation. The implementation of the OCR decoding head mainly includes three stages: (1) estimating a rough semantic segmentation result based on the feature representation of the middle layer of the network, (2) calculating the feature vector corresponding to the semantic category based on the rough semantic segmentation results and the deepest feature representation of the network, and (3) computing the relationship matrix between the pixel feature representation and the object region representation to obtain the final object contextual representation. Using the aforementioned method, the semantic category of each pixel can be predicted based on the augmented feature representation. This network utilizes cross-entropy as the loss function, and the weights of the C and OCR decoding heads are 0.4 and 1.0, respectively.

## Results

We conduct experiments utilizing the proposed compliant nano-manipulator-based SPL system and framework to optimize the process conditions and parameters of nano-lithography. We hereby present the results of the semantic segmentation network and the optimization of processing conditions and parameters.

### Data preparation for ML

We utilize the m-SPL system in tapping mode as our experimental example for analysis (the results of b-SPL and o-SPL are also validated in Section S8 of the Supplementary Information). The driving amplitude of the probe and scanning speed are identified as critical factors that affect the machining results while maintaining constant values for other conditions. We select 11 values for each of these two parameters to form 121 parameter pairs for the experiment. Setpoint, which denotes the amplitude, is chosen from 0.5 to 5.5 nA with an increment of 0.5 nA. Similarly, the scanning speed values are chosen as 0.5, 1, 5, 10, 50, 100, 300, 500, 1000, 2000, and 3560 μm/s, respectively. To minimize error, nine lines are produced under each parameter pair, and the processing range is 1 × 1 μm^2^ with a parallel line spacing of 100 nm. After processing, we use the same probe to acquire the surface topography, and the sample is scanned at a frequency of 1 Hz with an AFM image resolution of 256 × 256. To avoid variations in the experimental results due to the tip size of different probes, we conducted all experiments using the same probe. Our results indicate that there is no significant difference in image quality across all 121 sets of experimental results, which suggests that the probe is not worn.

**Fig. 5:**
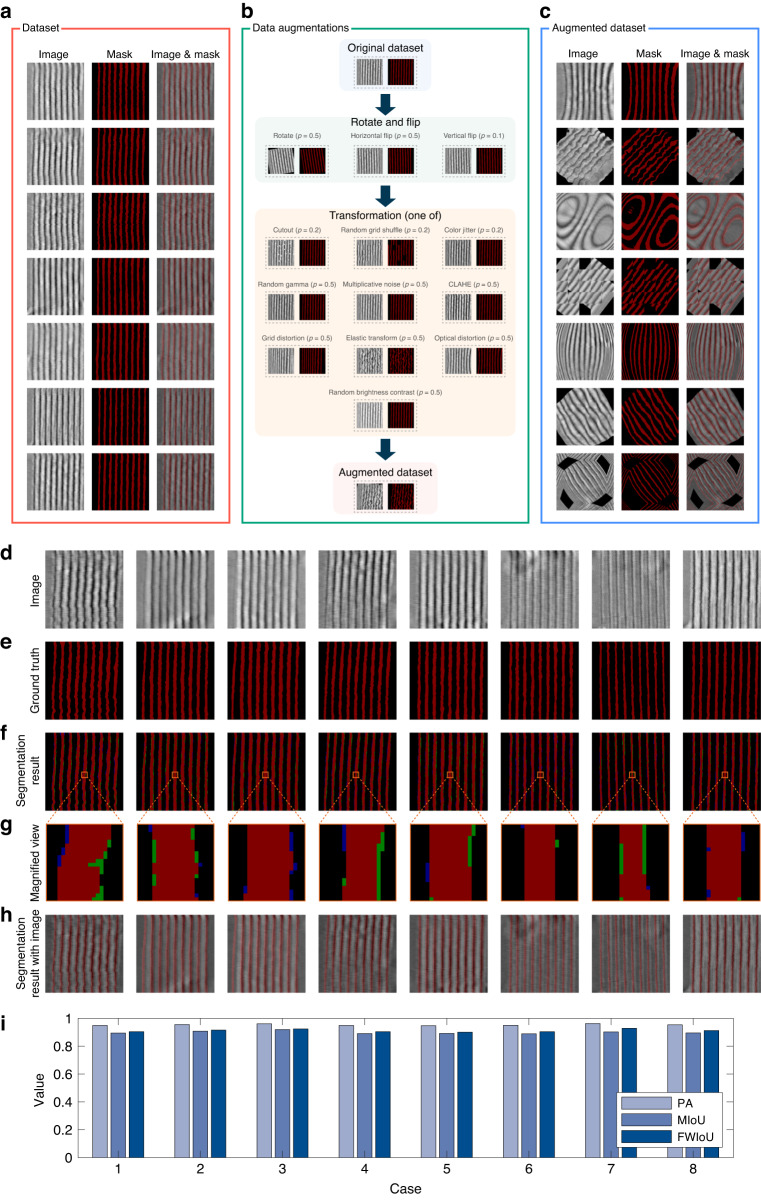
Dataset for AFM topology images and segmentation results of nano-structures. **a** Samples of images and masks in the dataset of AFM topology images. **b** Flow chart of the data augmentation method. **c** Augmented dataset. **d** Original AFM topology images in test set. **e** Ground truth corresponding to the original AFM topology images. **f** Segmentation results (true positive, false positive, false negative and true negative are marked in red, green, blue and black, respectively). **g** Magnified view of the segmentation result. **h** Segmentation results with original AFM topology images. **i** Histogram of semantic segmentation metrics for partial instances of the test set

For the purpose of training the network, the AFM topography images and their corresponding masks are required as inputs. In this task, we mainly split the lithography area from the background. To minimize labeling errors, we utilize the data preprocessing method detailed in Section “Prototype system, experiments and data preprocessing” while manually preparing the labels. In the masks, we assign the background area with black color and the FWHM area of the nano-structures with red color, as illustrated in Fig. 5a. And the dataset is then divided into three subsets, namely the training set, validation set, and test set, in a ratio of 80%, 10%, and 10%, respectively.

In order to effectively address the issue of overfitting in this few-shot learning task, data augmentation is performed on the training set. The process flow chart for this augmentation method is illustrated in Fig. 5b. Initially, the original dataset is rotated and flipped, and then one of ten transformations is applied to the dataset, which is then added to the augmented dataset. The probability (*p*) for each data augmentation method is depicted in Fig. 5b. Each data is randomly augmented five times. It should be noted that the corresponding masks are also augmented by using the same method. The part of resulting augmented dataset for AFM topology images of nano-structures is presented in Fig. 5c.

### Training of the HR-C-OCR semantic segmentation network

All training and validation procedures are implemented with the PyTorch package (GPU version 1.10.0). A total of 576 images are used for training, after data augmentation. A pre-trained model is utilized to improve the convergence speed of the algorithm. The model is trained for a total of 40,000 iterations and the batch size is set to 32. The stochastic gradient descent (SGD) optimizer is used with a learning rate of 0.01, momentum of 0.9, and weight decay of 0.0005. Training and deployment of HR-C-OCR network are conducted on a 64-bit workstation equipped with 32 GB RAM, an AMD Ryzen^TM^ 9 5900X CPU (12-core, 3.70 GHz) and an NVIDIA® GeForce GTX 3080 GPU.

### Performance of the HR-C-OCR semantic segmentation network

Based on the results of the HR-C-OCR semantic segmentation network and training process, we verified the effectiveness of the method in the test set, and some results are shown in Fig. 5. The original AFM topology and its corresponding ground truth are shown in Fig. 5d and Fig. 5e, respectively. In Fig. 5f, we present the segmentation result achieved using the HR-C-OCR network, with an enlarged view provided in Fig. 5g. Furthermore, Fig. 5h shows segmentation results using the original AFM topology images. It is noteworthy that the false positives and false negatives are primarily observed at the edges of the nano-structures. Some of these inaccuracies may arise from manual labeling errors instead of segmentation errors of the network.

To further assess the performance of the semantic segmentation network, a total of eight test images are selected for the purpose of quantifying the segmentation outcomes. The pixel accuracy (PA), the mean intersection over union (MIoU), as well as the frequency weighted intersection over union (FWIoU) are crucial metrics that are utilized to evaluate the results of the semantic segmentation of images. The values of these metrics for the selected cases are presented in Fig. 5i. The above analytical metrics demonstrate that the proposed approach can effectively segment the nano-structures for subsequent analysis and processing. Additional details and evaluation metrics concerning the segmentation outcomes for nano-structures can be found in the Supplementary Information (see Section S5 of the Supplementary Information for the details of the performance evaluation of the proposed semantic segmentation network).

### Statistical results of nano-structures

**Fig. 6:**
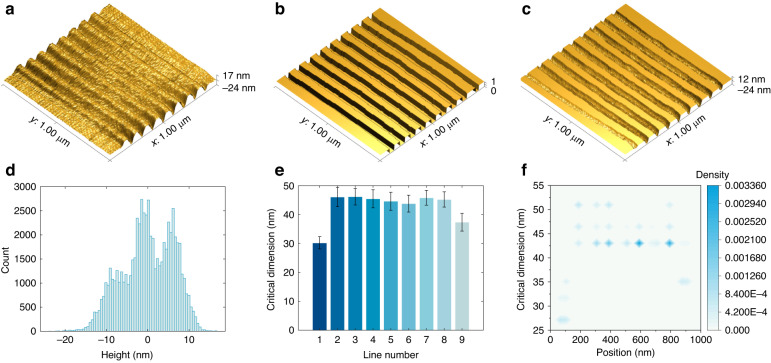
Statistical and analytical results of the nano-structures. **a** The AFM topology image of nano-structures (1000 nm × 1000 nm). **b** Segmentation results of the AFM topology image based on the ML network (1000 nm × 1000 nm, 0: nano-structures, 1: background). **c** The AFM topology image of nano-structures below the FWHM section with the segmentation results (1000 nm × 1000 nm). **d** Statistical histogram of the height of the nano-structures. **e** Histogram of the critical dimensions of different nano-structures. **f** 2D density distribution at positions and critical dimensions of different nano-structures

The proposed framework allows for the automatic processing of AFM topological images using ML to extract a multitude of information. Here we show a nano-lithography result as an example to demonstrate the credible and comprehensive statistical and analytical results automatically obtained based on the framework, as shown in Fig. 6.

Figure 6a, b show the AFM topology image of the nano-structures and their segmentation results obtained through the proposed framework, respectively. By utilizing the segmentation results, it is possible to obtain the AFM topology image of the nano-structures located beneath the FWHM section, as illustrated in Fig. 6c. Based on the AFM topology image and segmentation results, multiple statistical and analytical results can be easily obtained for various parameters. Figure 6d portrays the statistical histogram of the height of the nano-structures in the AFM topology image. In the nano-structures fabricated using the proposed compliant nano-manipulator-based SPL system, critical dimension and position are important parameters. To illustrate, we present the statistical results of the critical dimensions at different positions of all nano-structures in Fig. 6e. Moreover, Fig. 6f illustrates the density distribution relationship between critical dimension and position. These statistics can be used as standard evaluation indicators to adjust and optimize the manufacturing process to achieve the desired manufacturing quality under appropriate processing conditions and parameters.

### Nano-lithography with optimization

**Fig. 7:**
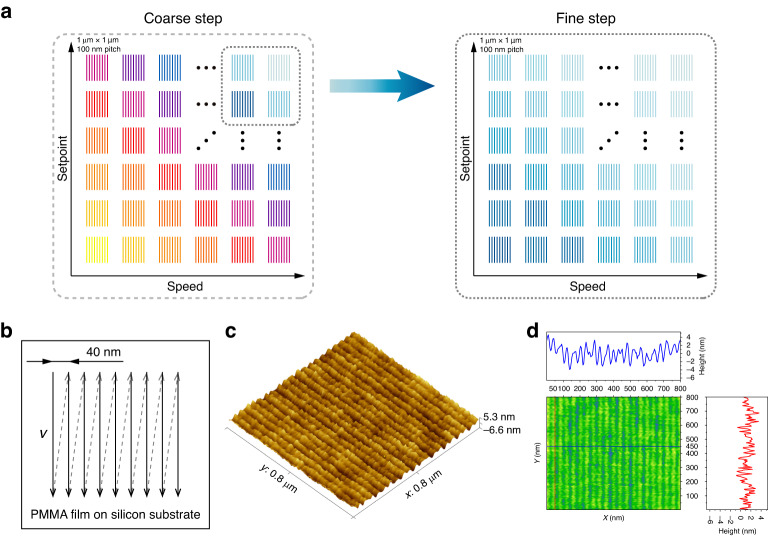
Process parameter optimization method and experimental results of nano-lithography. **a** Coarse-to-fine nano-lithography process method. **b** The trajectory of the AFM probe tip. **c** The AFM topology image of nano-structures. The scale bar represent 40 nm. **d** AFM topography image of the nano-structures with cross-sectional lines

Based on the proposed nano-lithography framework illustrated in Fig. 3 and the ML-based statistical evaluation result, we optimized the processing conditions and parameters, as shown in Fig. 7. For nano-lithography with multiple process parameters, it is necessary to conduct a large number of experiments in the space formed by these parameters to find the optimal process parameters. However, this approach can be very time-consuming, and it may not be possible to test all the parameters. To overcome these challenges, we propose a coarse-to-fine nano-lithography process method, as illustrated in Fig. 7a. To introduce the method, we consider the two process parameters of setpoint and speed in m-SPL as examples. The proposed method is divided into two steps, namely coarse step and fine step. In the coarse step, the value range of the selected parameters, such as the scanning speed and the setpoint, is roughly divided. After nano-lithography, the same probe is used for in-situ characterization. With the help of the proposed ML-based framework, a variety of statistical analysis data of the preparation results can be obtained. The nano-lithography results are then utilized to obtain the local range of parameters corresponding to the desired manufacturing results. Next, the above local range of parameters is finely divided to find the optimal parameter. In each group of experiments, multiple features are considered to represent the fabrication condition to minimize the interference of random factors (see Section S7 of the Supplementary Information for the details of the experimental results of the coarse-to-fine nano-lithography process method).

Using the aforementioned coarse-to-fine nano-lithography process method, we optimized the process parameters to achieve reduced critical dimensions. Note that the previous nano-structures are all 100 nm pitches with a critical dimension of about 45 nm, as shown in Fig. 6e. The spacing between parallel lines is typically greater than twice the critical dimension to ensure stable and repeating nano-structures. By optimizing the process parameters, we reduce the spacing between the parallel lines from 100 nm to 40 nm, as depicted by the trajectory of the AFM probe tip in Fig. 7b. The experimental results demonstrate that we successfully fabricated nano-structures with a pitch of 40 nm and a critical dimension of approximately 17 nm, as illustrated in Fig. 7c, d. A smaller critical dimension facilitates to realize the fabrication of nano-structures with fine features. The proposed framework and automatic evaluation of nano-lithography results accelerate the optimization process of process conditions and parameters.

### Large-area nano-lithography

**Fig. 8:**
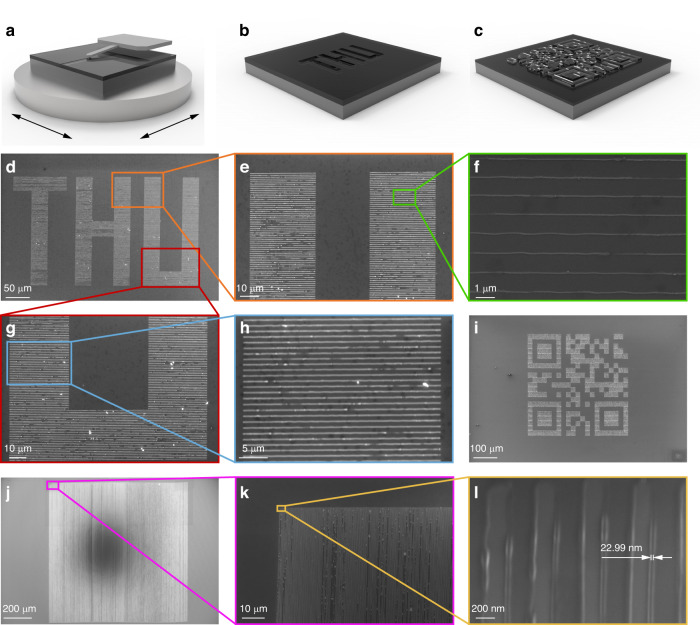
Experiment results of large-area m-SPL. **a** The scanning probe, sample and motion stage. **b** Schematic diagram of a sample lithographed with large-area letters “THU''. **c** Schematic diagram of a sample lithographed with a large-area QR code. **d** SEM image of the large-area letters “THU” (386 × 229 μm^2^). **e** Partial enlarged view of **d**. **f** Partial enlarged view of **e**. **g** Partial enlarged view of **d**. **h** Partial enlarged view of **g**. **i** SEM image of the large-area QR code (420 × 420 μm^2^). **j** SEM image of the large-area line arrays (1 × 1 mm^2^). **k** Partial enlarged view of **j**. **l** Partial enlarged view of **k**

Further, we performed large-area nano-lithography based on the process parameters determined by the proposed optimization framework, as shown in Fig. 8. In this experiment, the motion stage of the compliant nano-manipulator drives the sample to perform a large-area continuous planar motion, as shown in Fig. 8a. And the motion trajectory is determined by the pattern to be fabricated. The probe is in contact with the sample surface and applies force to realize nano-lithography. Figure 8b, c illustrate the schematic diagrams of the large-area patterns consisting of the letters “THU” and a QR code encoded the letters “TSINGHUA” by nano-lithography, respectively. The sample is a silicon wafer spin-coated with PMMA. And the pattern is composed of a large amount of nanolines. Figure 8d shows the scanning electron microscope (SEM) image of a large-area sample with letters “THU” created using nano-lithography. The pattern consists of nanolines with a pitch of 1 μm, and its overall size is 386 × 229 μm^2^. The speed of nano-lithography is 100 μm/s, and it takes a total of 26 minutes and 26 seconds. Figure 8e–h show the partial enlarged views of the pattern. Additionally, Fig. 8i demonstrates the SEM image of the pattern of the QR code, with an overall size of 420 × 420 μm^2^. The speed of the nano-lithography process is 200 μm/s, and it takes a total of 76 minutes and 47 seconds (see Section S9 of the Supplementary Information for the details of the large-area nano-lithography). Furthermore, Fig. 8j–l show the SEM images of the line array with an area of 1 × 1 mm^2^ and a pitch of 500 nm. The critical dimension of the nano-structure is about 22.99 nm, as shown in Fig. 8l. The speed of nano-lithography is 1 mm/s, and it takes a total of 86 minutes and 43 seconds. Compared with the stitched method, the stitchless nano-lithography result demonstrates an absence of stitching error, and the throughput is increased by 48% (see Section S10 of the Supplementary Information for the details of the results with the stitched method and the effect of long-time SPL on accuracy). We also compare different nano-lithography methods (see Section S13 of the Supplementary Information for the details of the comparisons of different nano-lithography methods). These nano-lithography results demonstrate that our proposed system and ML-based framework enable large-area, stitchless, and high-efficiency direct-write nano-fabrication and information-encoded storage.

## Conclusion

In this paper, we have proposed a smart SPL, namely a framework accelerating nano-fabrication process with in-situ characterization via ML. The HR-C-OCR network has been employed in the proposed framework to segment the nano-structures, followed by an automated statistical analysis of the nano-lithography results. Furthermore, the critical dimension of the fabricated nano-structures has been reduced by 62% using the optimized process parameter based on the proposed framework and the results of statistical analysis. Moreover, large-area patterns with a size of hundreds of microns are directly-written based on the proposed ML-based framework. The experimental results confirm that the proposed framework accelerates the optimization of process conditions and parameters in the nano-fabrication process.

### Supplementary information

Supplemental Material
