# Supplementary material for: Towards smart scanning probe lithography: a framework accelerating nano-fabrication process with in-situ characterization via machine learning
Source: Microsyst Nanoeng. 2023 Oct 10;9:128. doi: 10.1038/s41378-023-00587-z (PMC10564742; doi:10.1038/s41378-023-00587-z)
Supplement: Supplementary file 1 — Supplemental Material [file 41378_2023_587_MOESM1_ESM.pdf]

Supplementary Information
Towards smart scanning probe lithography: A framework accelerating nano-fabrication process with in-situ characterization via machine learning

Yijie Liu[1,2] Xuexuan Li[1,2] Ben Pei[3,4,5] Lin Ge[6]
Zhuo Xiong[3,4,5,*] Zhen Zhang[1,2,*]

[1]State Key Laboratory of Tribology in Advanced Equipment, Department of Mechanical Engineering, Tsinghua University, Beijing, 100084, China.
[2]Beijing Key Laboratory of Precision/Ultra-precision Manufacturing Equipments and Control, Tsinghua University, Beijing, 100084, China.
[3]Biomanufacturing Center, Department of Mechanical Engineering, Tsinghua University, Beijing, 100084, China.
[4]Biomanufacturing and Rapid Forming Technology Key Laboratory of Beijing, Beijing, 100084, China.
[5]'Biomanufacturing and Engineering Living Systems' Innovation International Talents Base (111 Base), Beijing, 100084, China.
[6]NT-MDT Spectrum Instruments China office, Beijing, 100053, China.
*To whom correspondence should be addressed. E-mail:xiongzhuo@tsinghua.edu.cn (Z. Xiong) and zzhang@tsinghua.edu.cn (Z. Zhang)

Contents

Supplementary Note 1	Design of the XY compliant nano-manipulator	2
Supplementary Note 2	Modelling of the CNM	2
Supplementary Note 3	Finite element analysis of the CNM	6
Supplementary Note 4	Prototype of the CNM	6
Supplementary Note 5	Performance evaluation of the proposed semantic segmentation network	8
Supplementary Note 6	Comparison of traditional SPL process parameter optimization method and the proposed one	9
Supplementary Note 7	Experimental results of the coarse-to-fine nano-lithography process method	12
Supplementary Note 8	Experimental results of b-SPL and o-SPL	15
Supplementary Note 9	Details of large-area nano-lithography	15
Supplementary Note 10	Stitchless and stitched large-area SPL	15
Supplementary Note 11	SPL results on hard substrates	22
Supplementary Note 12	Scanning probe in the SPL system	23
Supplementary Note 13	Comparisons of different nano-lithography methods	25

Supplementary Note 1 Design of the XY compliant nano-manipulator

Compliant nano-manipulators (CNMs) are well suited for the applications with nanometric precision thanks to their features such as friction-free, maintenance-free and compact desktop-size. The desired compliant nano stages should have the following characteristics: nanometric motion quality (< 100 nm) [S1], large stroke (> 1 mm) [S1–S3], high speed [S3, S4], small parasitic rotation [S2, S3], small cross-axis coupling [S5, S6], and high linearity [S3]. It is very challenging for the existing compliant XY motion stages to achieve nanometric precision, large range and other good features simultaneously. Although considerable advances have been made, it is still difficult to achieve both nanometric precision and macro range for a planar layout leaf-spring based motion stage through comprehensively considering parasitic rotation, cross-axis coupling and appropriate natural frequencies.

A conceptual design of the XY CNM is provided to achieve nanometric precision and large range. As shown in Fig. S1a, the manipulator consists of the guiding mechanism, the redundant constraint one, the decoupling one and the motion stage. The dimensions of each leaf spring are labeled in Fig. S1b.

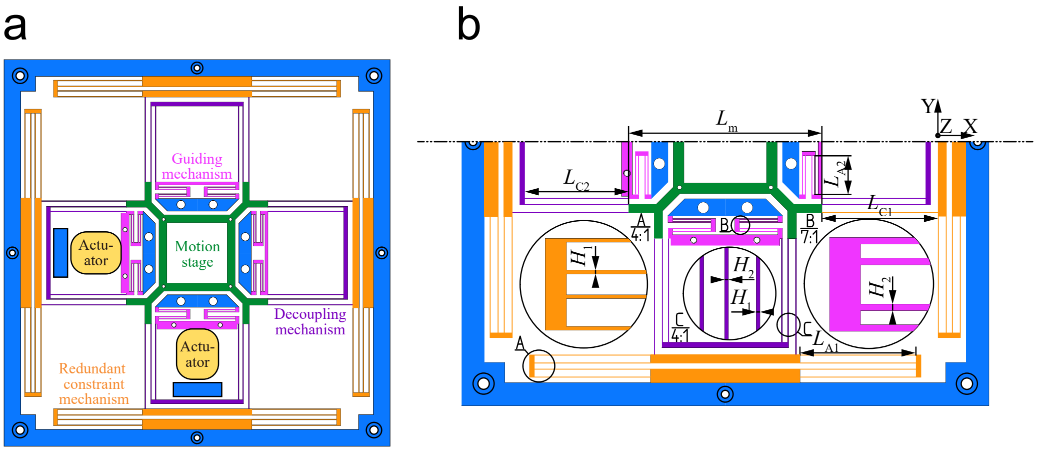

Figure S1: A conceptual design of an XY CNM. **a** Top view. **b** Top view with labeled geometric parameters.

Based on the design and modelling, the size parameters of the CNM are determined. The overall size of the compliant manipulator is 300 mm × 300 mm × 14 mm, which keeps the manipulator desktop-level. The geometric parameters and values are shown in Tab. S1.

Table S1: Geometric dimensions of the XY CNM

Parameters	L_{A1}	L_{A2}	L_{C1}	L_{C2}	L_t	H_1	H_2	B
Values (mm)	66.00	22.00	66.00	59.00	110.00	0.50	0.52	12.00

Supplementary Note 2 Modelling of the CNM

With the above design, we need to model the proposed compliant manipulator to determine its static and dynamic features. By design, the linearity of the load-displacement

curve is usually good within the travel range, and hence the stiffness matrix model can be adopted in the static analysis [S7].

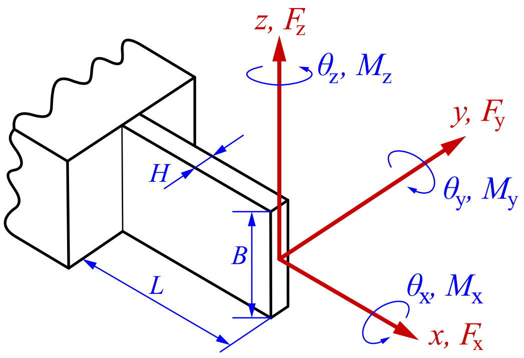

Figure S2: Coordinates of the flexure beam.

Fig. S2 shows a flexure beam with its coordinates. The forces and moments in/around certain axes are exerted on certain points, and the relationship between displacements and loads are formulated as follows,

$$\mathbf{X} = \mathbf{CF} \tag{1}$$
$$\mathbf{F} = \mathbf{KX} \ , \tag{2}$$

where $\mathbf{F} = [F_\mathrm{x}, F_\mathrm{y}, F_\mathrm{z}, M_\mathrm{x}, M_\mathrm{y}, M_\mathrm{z}]^\mathrm{T}$ and $\mathbf{X} = [\delta_\mathrm{x}, \delta_\mathrm{y}, \delta_\mathrm{z}, \theta_\mathrm{x}, \theta_\mathrm{y}, \theta_\mathrm{z}]^\mathrm{T}$ are the sets of forces/moments and translational displacements/rotational angles, respectively, and $\mathbf{K}$ is the stiffness matrix, and the compliance matrix $\mathbf{C} = \mathbf{K}^{-1}$ is of the following form

$$\mathbf{C} = \begin{bmatrix} \frac{L}{EBH} & 0 & 0 & 0 & 0 & 0 \\ 0 & \frac{4L^3}{EBH^3} & 0 & 0 & 0 & \frac{6L^2}{EBH^3} \\ 0 & 0 & \frac{4L^3}{EB^3H} & 0 & -\frac{6L^2}{EB^3H} & 0 \\ 0 & 0 & 0 & \frac{L}{Gk_2B^3H} & 0 & 0 \\ 0 & 0 & -\frac{6L^2}{EB^3H} & 0 & \frac{12L}{EB^3H} & 0 \\ 0 & \frac{6L^2}{EBH^3} & 0 & 0 & 0 & \frac{12L}{EBH^3} \end{bmatrix} . \tag{3}$$

The compliance matrix $\mathbf{C}_\mathrm{j}$ at coordinate $xo_\mathrm{i}y$ can be obtained by transforming the compliance matrix $\mathbf{C}_\mathrm{i}$ at the local coordinate $xo_\mathrm{i}y$

$$\mathbf{C}_\mathrm{j} = \mathbf{R}(\theta_\mathrm{i})\mathbf{P}(r_\mathrm{i})\mathbf{C}_\mathrm{i}\mathbf{P}(r_\mathrm{i})^\mathrm{T}\mathbf{R}(\theta_\mathrm{i})^\mathrm{T} \ , \tag{4}$$

where matrix $\mathbf{P}(r_\mathrm{i})$ is the transformation matrix from the local coordinate to a global coordinate, matrix $\mathbf{R}(\theta_\mathrm{i})$ is the direction transformation matrix. Matrices $\mathbf{P}(r_\mathrm{i})$ and $\mathbf{R}(\theta_\mathrm{i})$ can be expressed as follows

$$\mathbf{P}(r_\mathrm{i}) = \left[\begin{array}{c:ccc} \mathbf{I}_{3\times3} & \begin{matrix} 0 & -z_\mathrm{i} & y_\mathrm{i} \\ z_\mathrm{i} & 0 & -x_\mathrm{i} \\ -y_\mathrm{i} & x_\mathrm{i} & 0 \end{matrix} \\ \hdashline \mathbf{O}_{3\times3} & \mathbf{I}_{3\times3} \end{array}\right] , \tag{5}$$

where vector $r_\mathrm{i} = [x_\mathrm{i}, y_\mathrm{i}, z_\mathrm{i}]^\mathrm{T}$ is the one from the local coordinate o_i to the global coordinate o_j, and

$$\mathbf{R}(\theta_\mathrm{i}) = \left[\begin{array}{c:c} \widetilde{\mathbf{R}}(\theta_\mathrm{i}) & \mathbf{O}_{3\times3} \\ \hdashline \mathbf{O}_{3\times3} & \widetilde{\mathbf{R}}(\theta_\mathrm{i}) \end{array}\right] \tag{6}$$

$$\widetilde{\mathbf{R}}(\theta_\mathrm{i}) = \mathbf{R}_\mathrm{x}(\theta_\mathrm{ix}) \cdot \mathbf{R}_\mathrm{y}(\theta_\mathrm{iy}) \cdot \mathbf{R}_\mathrm{z}(\theta_\mathrm{iz}) \tag{7}$$

$$\mathbf{R}_\mathrm{x}(\theta_\mathrm{ix}) = \begin{bmatrix} 1 & 0 & 0 \\ 0 & \cos\theta_\mathrm{ix} & -\sin\theta_\mathrm{ix} \\ 0 & \sin\theta_\mathrm{ix} & \cos\theta_\mathrm{ix} \end{bmatrix} \tag{8}$$

$$\mathbf{R}_\mathrm{y}(\theta_\mathrm{iy}) = \begin{bmatrix} \cos\theta_\mathrm{iy} & 0 & \sin\theta_\mathrm{iy} \\ 0 & 1 & 0 \\ -\sin\theta_\mathrm{iy} & 0 & \cos\theta_\mathrm{iy} \end{bmatrix} \tag{9}$$

and

$$\mathbf{R}_\mathrm{z}(\theta_\mathrm{iz}) = \begin{bmatrix} \cos\theta_\mathrm{iz} & -\sin\theta_\mathrm{iz} & 0 \\ \sin\theta_\mathrm{iz} & \cos\theta_\mathrm{iz} & 0 \\ 0 & 0 & 1 \end{bmatrix}. \tag{10}$$

The compliance matrix method is used to model the CNM. The redundant constraint mechanism is a quadruple parallelogram module, the minimum unit of the module is modeled, and then the compliance matrix of the whole module is obtained with the transformation and superposition of the compliance matrix. Fig. S3a shows the minimum unit - the parallelogram module, and Fig. S3b presents the right half of the whole module, and Fig. S3c displays the whole module.

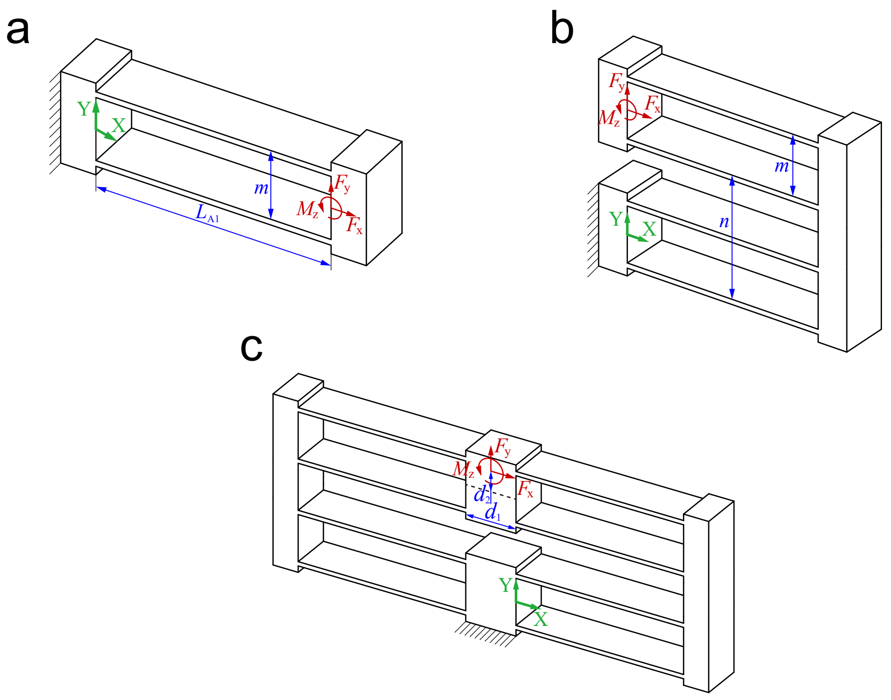

Figure S3: Schematic diagram and coordinates of compliant mechanisms. **a** Parallelogram module. **b** Double parallelogram module. **c** Quadruple parallelogram module.

Based on the compliance matrix of the flexure beam, the stiffness matrix of the parallelogram module is given by

$$\mathbf{K}_{\mathrm{p}} = \mathbf{C}_{\mathrm{p1}}^{-1} + \mathbf{C}_{\mathrm{p2}}^{-1} \ , \tag{11}$$

where $\mathbf{C}_{\mathrm{p1}}$ and $\mathbf{C}_{\mathrm{p2}}$ are the compliance matrices of the two beams, respectively.

Hence the compliance matrix of the double parallelogram module can be calculated by

$$\mathbf{C}_{\mathrm{d}} = \sum_{\mathrm{i=1}}^{2} \mathbf{R}(\theta_{\mathrm{di}})\mathbf{P}(r_{\mathrm{di}})\mathbf{C}_{\mathrm{p}}\mathbf{P}(r_{\mathrm{di}})^{\mathrm{T}}\mathbf{R}(\theta_{\mathrm{di}})^{\mathrm{T}} \ , \tag{12}$$

where θ_{di} and r_{di} are the transformation angles and transformation vectors, respectively.

Thus, the compliance matrix of the quadruple parallelogram module is given by

$$\mathbf{K}_{\mathrm{q}} = \mathbf{C}_{\mathrm{d1}}^{-1} + \mathbf{K}_{\mathrm{d2}}^{-1} \ , \tag{13}$$

where $\mathbf{C}_{\mathrm{d1}}$ and $\mathbf{C}_{\mathrm{d2}}$ are the compliance matrices of the two double parallelogram modules, respectively.

Similarly, the stiffness matrix of the guiding mechanism $\mathbf{K}_{\mathrm{g}}$, the stiffness matrix of the parallelogram mechanism $\mathbf{K}_{\mathrm{p}}$, and the stiffness matrix of the double parallelogram mechanism $\mathbf{K}_{\mathrm{dp}}$ can be obtained. The compliance matrix of a quarter of the CNM can be expressed as follows,

$$\mathbf{C}_{\mathrm{S}} = (\mathbf{K}_{\mathrm{q}} + \mathbf{K}_{\mathrm{g}})^{-1} + (\mathbf{K}_{\mathrm{p}} + \mathbf{K}_{\mathrm{dp}})^{-1} \ . \tag{14}$$

Since the manipulator contains four identical parts, the total stiffness matrix of the manipulator $\widetilde{\mathbf{K}}_{\mathrm{S}}$ can be written as follows

$$\widetilde{\mathbf{K}}_{\mathrm{S}} = \sum_{\mathrm{i=1}}^{4} \left[\mathbf{R}(\theta_{\mathrm{i}})\mathbf{C}_{\mathrm{S}}\mathbf{R}(\theta_{\mathrm{i}})^{\mathrm{T}}\right]^{-1} \ , \tag{15}$$

where θ_{i} is the transformation angles.

By simplifying the elements of matrix $\widetilde{\mathbf{K}}_{\mathrm{S}}$, it can be found $\widetilde{\mathbf{K}}_{\mathrm{S1,1}} = \widetilde{\mathbf{K}}_{\mathrm{S2,2}}$, the axial stiffness in the X direction is the same as the one in the Y direction. The axial stiffness of the CNM is shown as follows,

$$K_{\mathrm{S}} = 2EB\left(\frac{2H_1^3}{L_{\mathrm{A1}}^3} + \frac{2H_2^3}{L_{\mathrm{A2}}^3} + \frac{2H_1^3}{L_{\mathrm{C1}}^3} + \frac{H_2^3}{L_{\mathrm{C2}}^3}\right) \ . \tag{16}$$

The CNM is simplified to a single-degree-of-freedom mass-stiffness system as shown in Eq. (17), of which the natural frequency is calculated by Eq. (18)

$$M_{\mathrm{S}}\ddot{x} + K_{\mathrm{S}}x = 0 \tag{17}$$

$$f_{\mathrm{S}} = \frac{1}{2\pi}\sqrt{\frac{K_{\mathrm{S}}}{M_{\mathrm{S}}}} \ , \tag{18}$$

where M_{S} is the equivalent mass of the stage.

Supplementary Note 3 Finite element analysis of the CNM

To validate the analytical model and the comprehensive performance of the CNM, the finite element analysis (FEA) is implemented using the software ANSYS®.

According to the static model shown in Eq. (16), the theoretical relationship between the axial force and displacement are plotted in Fig. S4 (solid line). For comparison, axial forces ($F_x = F_y =$ [5 10 15 20 25 30 35 40 45 50] N) are applied to the actuators of the X axis and Y axis respectively to simulate the displacement of the motion stage in ANSYS®. The simulation results are shown in Fig. S4 (circle and square marks), which agree well with the theoretical model.

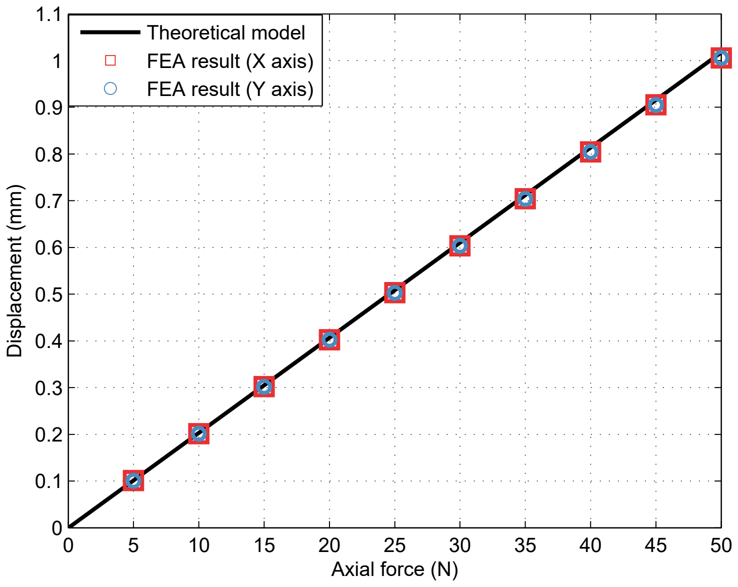

Figure S4: Axial stiffness analysis results

The errors between the simulation results of axial stiffness and the theoretical model of the X axis and Y axis are 1.17 % and 1.13 %, respectively. The simulation results further verify the validity of linear stiffness matrix model.

The dynamic features of the compliant manipulator are investigated with the modal analysis. And the results are shown in Fig. S5, where the first and second modals represent the translation of the motion stage in the X and Y axis, respectively. The first and second natural frequencies are 60.57 Hz and 60.68 Hz, respectively, which are significantly higher than those of the existing leaf-spring based compliant manipulators. Comparing to the natural frequency calculated by Eq. (18), the errors between theoretical dynamic model and FEA simulation are less than 1%.

Supplementary Note 4 Prototype of the CNM

Fig. S6 shows the compact prototype of the large range CNM. The prototype is monolithically fabricated with aluminium alloy 7075-T6 via wire electric discharge machining. The base of the CNM is fabricated with cast iron to provide vibration isolation. Two moving magnet voice coil motors are custom designed to actuate the manipulator with the peak force of 259 N and motion range of 8 mm. The translational displacements of

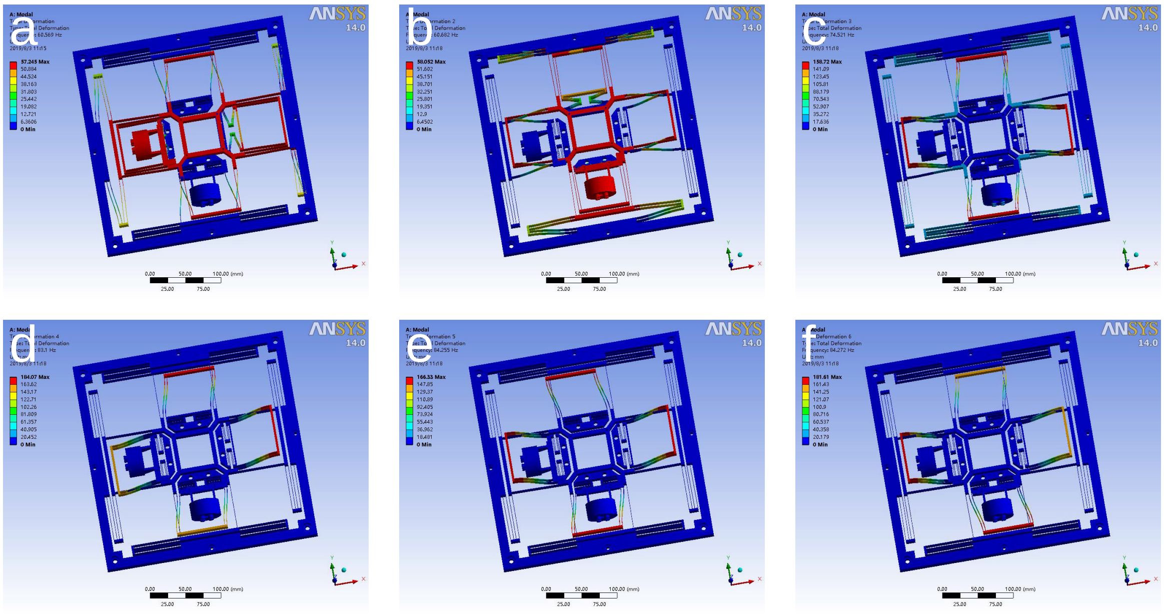

Figure S5: First six-order modal analysis of the CNM. **a** 1st modal (60.57 Hz). **b** 2nd modal (60.68 Hz). **c** 3rd modal (74.52 Hz). **d** 4th modal (83.10 Hz). **e** 5th modal (84.26 Hz). **f** 6th modal (84.27 Hz).

the motion stage are fed back by two linear optical encoders providing a resolution of 1.2 nm in the motion range.

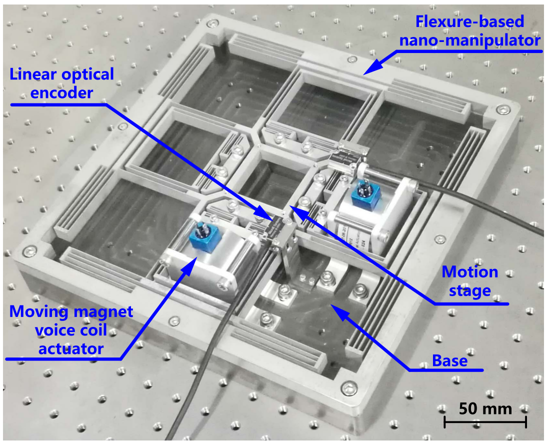

Figure S6: Prototype CNM.

As shown in Fig. S7, the real-time control is implemented by using the dSPACE-R1103 rapid prototyping system. The sampling time applied in the servo is 10 kHz. A laser interferometer (attocube IDS3010) is utilized to measure the displacement difference of two sides of the motion stage to calibrate the two linear optical encoders.

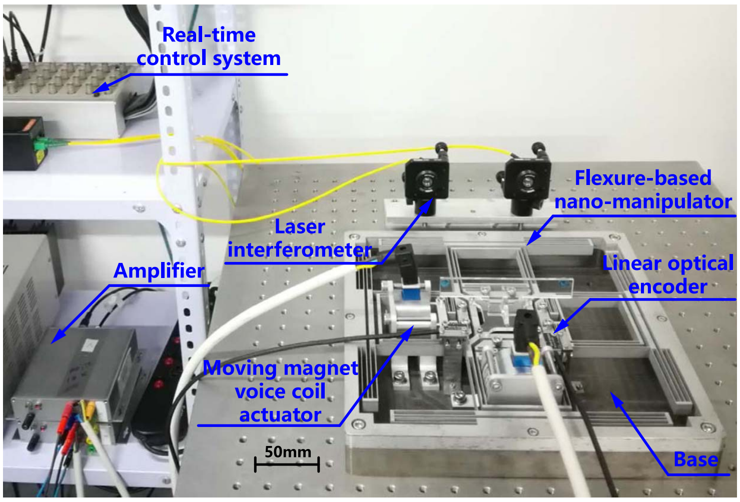

Figure S7: XY CNM system.

Supplementary Note 5 Performance evaluation of the proposed semantic segmentation network

To evaluate the effectiveness of the semantic segmentation network, we compute seven metrics for evaluation purposes. These metrics include pixel accuracy (PA), precision, average precision (AP), intersection over union (IoU), mean intersection over union (MIoU), frequency weighted intersection over union (FWIoU), and F_1-score (F_1). The PA metric indicates the percentage of pixels that are classified correctly in the image. Precision provides information about the purity of positive detections with respect to the ground truth. AP is the average precision across all classes. The IoU metric measures the intersection between the predicted and target masks, divided by the total number of pixels in both masks. The MIoU is the average IoU across all classes. FWIoU is the average weighted IoU based on the frequency of each class. The F_1 score represents the harmonic mean of precision and recall. The following are the definitions of each of these metrics:

$$\mathrm{PA} = \frac{\mathrm{TP}+\mathrm{TN}}{\mathrm{TP}+\mathrm{TN}+\mathrm{FP}+\mathrm{FN}}, \tag{19}$$

$$\mathrm{Precision} = \frac{\mathrm{TP}}{\mathrm{TP}+\mathrm{FP}}, \tag{20}$$

$$\mathrm{AP} = \frac{1}{k+1}\sum_{i=0}^{k}\frac{\mathrm{TP}}{\mathrm{TP}+\mathrm{FP}}, \tag{21}$$

$$\mathrm{Recall} = \frac{\mathrm{TP}}{\mathrm{TP}+\mathrm{FN}}, \tag{22}$$

$$\mathrm{IoU} = \frac{\mathrm{TP}}{\mathrm{TP}+\mathrm{FP}+\mathrm{FN}}, \tag{23}$$

$$\mathrm{MIoU} = \frac{1}{k+1}\sum_{i=0}^{k}\frac{\mathrm{TP}}{\mathrm{TP}+\mathrm{FP}+\mathrm{FN}}, \tag{24}$$

$$\mathrm{FWIoU} = \frac{\mathrm{TP}+\mathrm{FN}}{\mathrm{TP}+\mathrm{TN}+\mathrm{FP}+\mathrm{FN}}\frac{\mathrm{TP}}{\mathrm{TP}+\mathrm{FP}+\mathrm{FN}}, \tag{25}$$

$$F_1 = 2 \times \frac{\text{Precision} \times \text{Recall}}{\text{Precision} + \text{Recall}} = \frac{2\text{TP}}{2\text{TP} + \text{FP} + \text{FN}}, \tag{26}$$

where k is the number of categories of the segmentation target, TP, TN, FP and FN are true positive, true negative, false positive, and false negative, respectively.

To assess the quality of the segmentation results for each case presented in the test set, we compute the evaluation metrics outlined in Eqs. (19)–(26), and report the results in Tab. S2. The evaluation demonstrates that the proposed method successfully achieves precise segmentation of nano-structures, and the trained model can be readily utilized to optimize the conditions and parameters of the nano-lithography process.

Table S2: Evaluation metrics for segmentation result of nano-structures.

Metric / Case	PA	Precision		AP	IoU		MIoU	FWIoU	F_1	
		Bg[1]	Ns[2]		Bg	Ns			Bg	Ns
1	0.9491	0.9596	0.9294	0.9445	0.9248	0.8642	0.8945	0.9037	0.9609	0.9271
2	0.9555	0.9523	0.9614	0.9569	0.9330	0.8829	0.9079	0.9155	0.9653	0.9378
3	0.9603	0.9657	0.9516	0.9587	0.9377	0.9016	0.9196	0.9239	0.9678	0.9483
4	0.9490	0.9609	0.9242	0.9426	0.9271	0.8548	0.8909	0.9036	0.9622	0.9217
5	0.9469	0.9461	0.9485	0.9473	0.9204	0.8627	0.8915	0.9001	0.9585	0.9263
6	0.9495	0.9831	0.8789	0.9310	0.9296	0.8486	0.8891	0.9036	0.9635	0.9181
7	0.9620	0.9732	0.9266	0.9499	0.9511	0.8542	0.9026	0.9278	0.9749	0.9214
8	0.9537	0.9714	0.9119	0.9417	0.9365	0.8544	0.8954	0.9120	0.9672	0.9215

[1] Background
[2] Nano-structures

Supplementary Note 6 Comparison of traditional SPL process parameter optimization method and the proposed one

We detail the traditional SPL process parameter optimization method and the proposed one based on ML to show sufficient evidence demonstrating the benefits of the proposed framework.

Fig. S8 shows the flow chart of traditional SPL process parameter optimization method. First, the initial process parameters need to be given. In this example, we select two typical parameters, scan speed (v_i) and setpoint (s_i), for demonstration. After nano-lithography and in situ characterization, AFM images are obtained. Nano-fabrication results often exhibit variations across different locations, even when subjected to identical process parameters. We present the height distribution of two cross-sectional lines of the "Manual measurement" part in Fig. S8. It is evident that the width and depth of the same groove in nano-lithography vary at different locations. To describe the width of the nano-grooves, the parameter of full width at half maximum (FWHM) is usually adopted. The conventional approach is to select one or several cross-sectional lines for manual measurement of the FWHM of the nano-structures. However, it is very error-prone, time-consuming and usually difficult to fully characterize and measure the entire nano-structures manually. As a result, it remains challenging to optimize the process of nano-lithography without an accurate and reasonable metric. With the results of the manual measurement, the process parameters are adjusted for optimization. The inaccuracy associated with manual measurement and the lack of clear direction in adjusting process parameters significantly hinder the efficiency and results of the optimization process.

To tackle the above challenges, we propose the framework to accelerate nano-fabrication process with in-situ characterization via machine learning. Fig. S9 shows the flow chart of the proposed SPL process parameter optimization method.

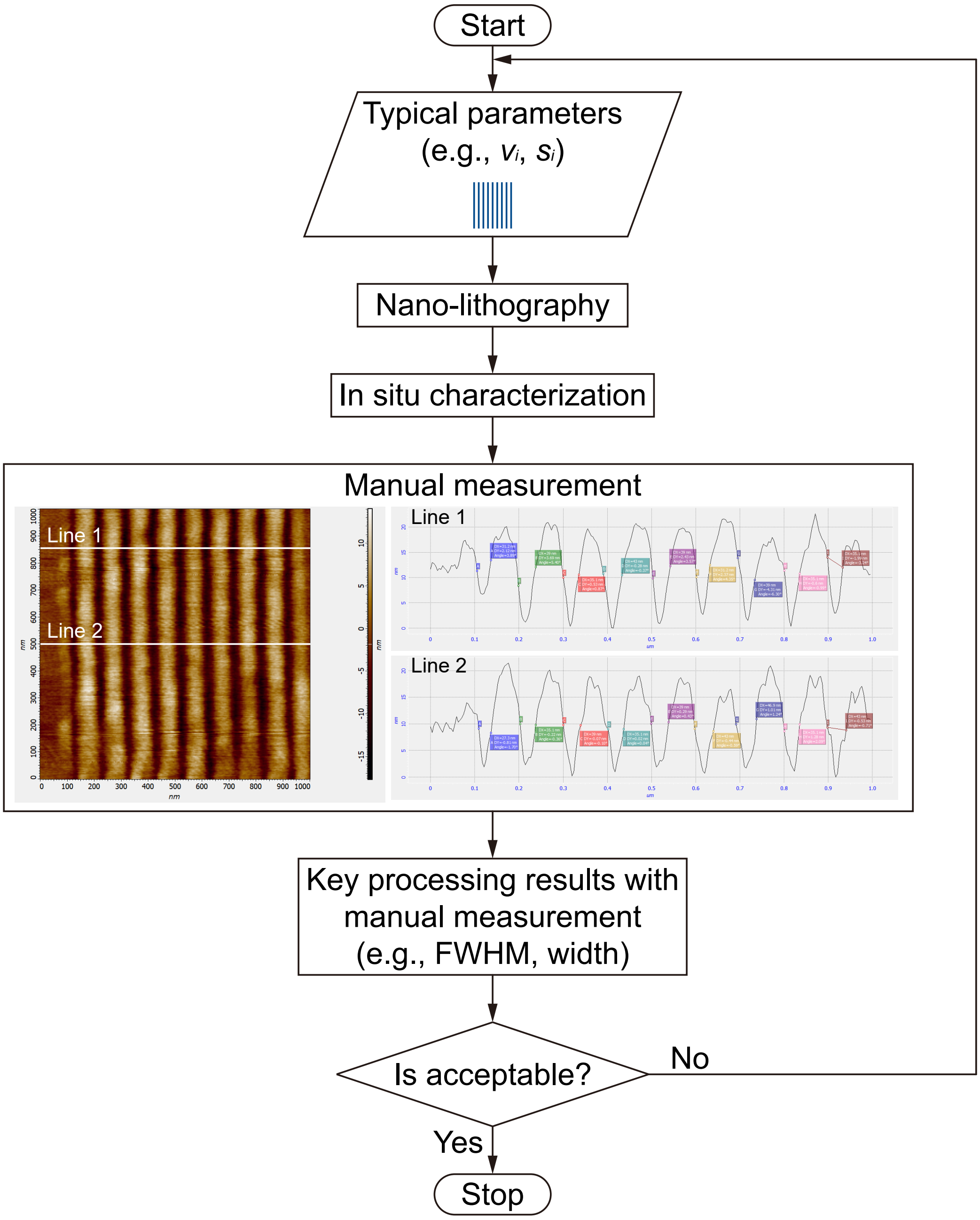

Figure S8: Flow chart of traditional SPL process parameter optimization method.

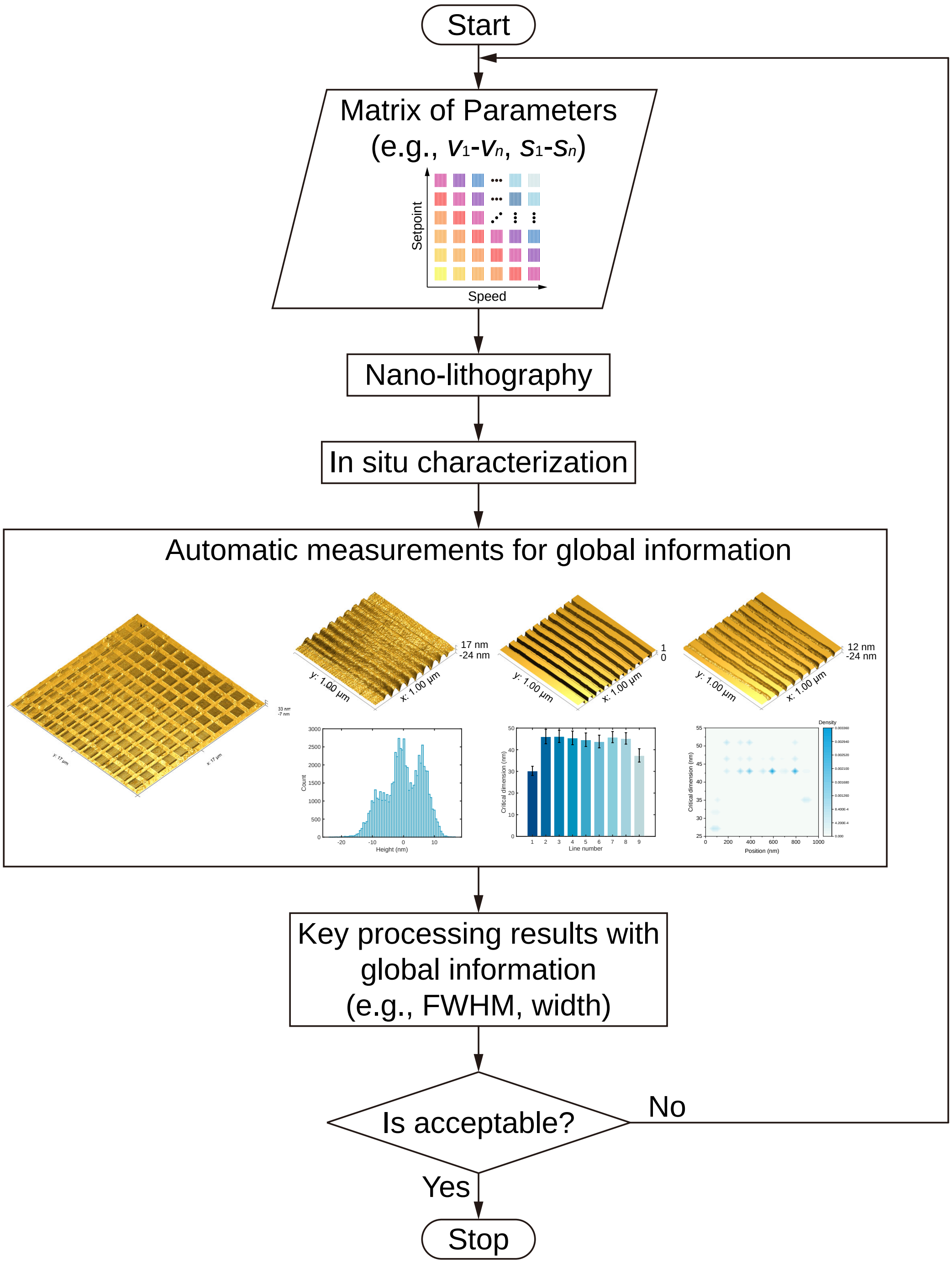

Figure S9: Flow chart of the proposed SPL process parameter optimization method.

In contrast to the conventional approach, which involves specifying two typical parameters, the proposed method employs a matrix of parameters. This facilitates rapid experimentation with multiple sets of parameters. The proposed framework allows for the automatic processing of AFM topological images using ML to extract a multitude of information. Here we show a nano-lithography result as an example to demonstrate the credible and comprehensive statistical and analytical results automatically obtained based on the framework, as shown in the "Automatic measurements for global information" part in Fig. S9. For each set of parameters, the information (e.g., FWHM and pitch) can be obtained automatically for each position in each etched area. Different from traditional SPL techniques that rely on manual labeling-based experimental methods, the proposed framework intelligently extracts reliable and global information for statistical analysis to fine-tune and optimize process parameters. The matrix of parameters is further refined to find the optimal parameters (coarse-to-fine nano-lithography process method).

Regarding additional process parameters, more experiments are usually required to complete the optimization process. Therefore, additional variables will complicate the optimization process. The proposed ML-based framework can still accelerate the optimization of multi-parameters with automatic and accurate measurement of nanofabrication results (e.g., FWHM and pitch). The proposed framework still has significant advantages in terms of efficiency and accuracy compared to process parameter optimization methods using manual measurement methods.

The proposed framework offers essential advantages and benefits, which can be summarized as follows:

★ The proposed ML-based framework focuses on the extraction of nano-structures and the automated calculation of important nano-fabrication results (e.g., FWHM and pitch). Specifically, global nano-fabrication results can be automatically obtained, which provides an accurate and fast measurement method for the process parameter optimization.

★ We also propose a coarse-to-fine nano-lithography process method for the rapid determination of optimal process parameters. The process of reducing pitch size relies on the FWHM and pitch information automatically obtained by the ML-based framework, which speeds up the optimization process of manual methods.

Supplementary Note 7 Experimental results of the coarse-to-fine nano-lithography process method

In the coarse-to-fine nano-lithography process method, we conduct experiments to obtain optimized process parameters. Specifically, the tapping mode of m-SPL is chosen for nano-lithography experimentation. The driving amplitude of the probe and scanning speed are identified as critical factors that affect the machining results while maintaining constant values for other conditions. For each of the aforementioned factors, 11 values are chosen, resulting in 121 parameter pairs for the experiment, which are illustrated in Fig. S10. The area of the nano-lithography pattern is 17×17 μm^2.

During the coarse step, the setpoint range is set from 0.5 to 5.5 nA, increasing at an interval of 0.5 nA. The scanning speed values, on the other hand, are selected as 0.5, 1, 5, 10, 50, 100, 300, 500, 1,000, 2,000, and 3,560 μm/s, respectively. To minimize error, nine lines are generated under each parameter pair, with each parameter being processed within a range of 1×1 μm^2 and a parallel line spacing of 100 nm. After processing, we use the same probe to acquire surface topography data. The sample is scanned at a

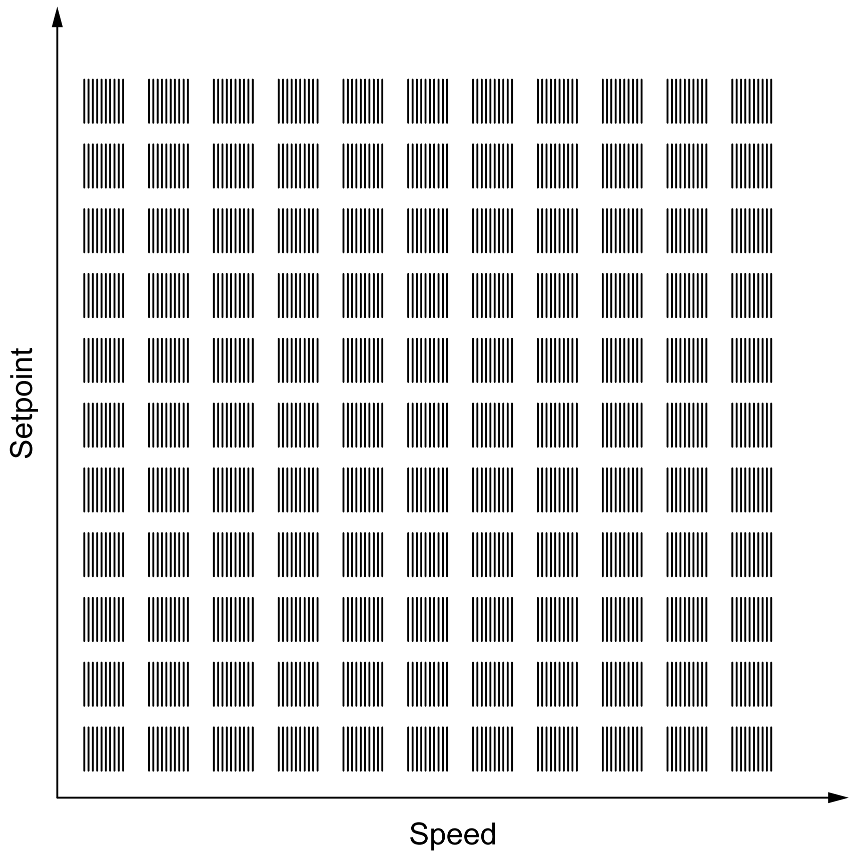

Figure S10: Lithography pattern in the coarse-to-fine process method.

frequency of 1 Hz, with an AFM image resolution of 1000 × 2000.

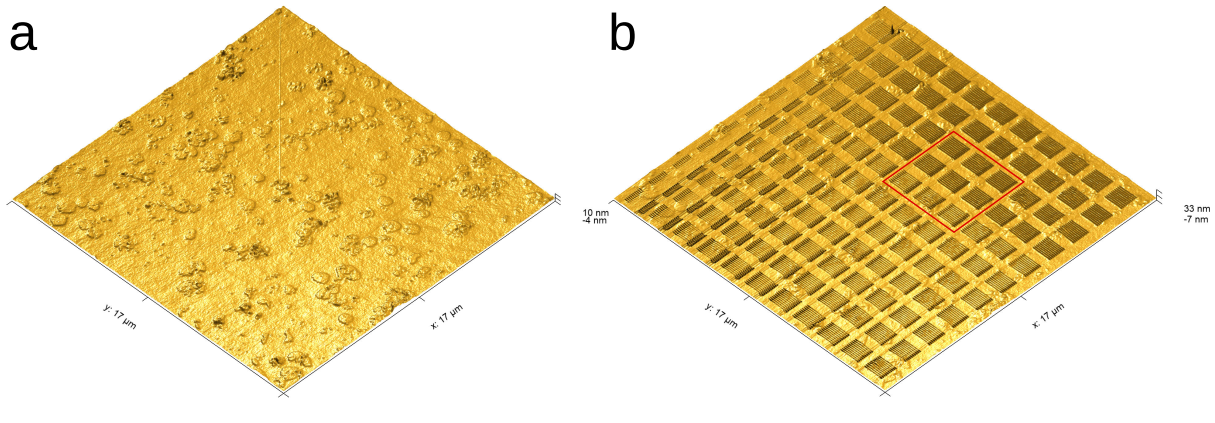

Figure S11: The AFM topology images in the coarse step of the process method. **a** Before nano-lithography. **b** After nano-lithography.

Fig. S11 presents the AFM topology image obtained during the coarse step of the process method. Fig. S11a showcases the topography of the sample surface after being spin-coated with PMMA before nano-lithography. Following nano-lithography and in-situ characterization, the resulting nano-structures are depicted in Fig. S11b. It can be observed that the critical dimension and depth of the nano-structures vary in a gradient with the change of the nano-lithography parameters. In this regard, we select the region of

interest and fine-tune its parameters to determine the optimized process parameters. The red box illustrated in Fig. S11b highlights the nano-structures of the region of interest.

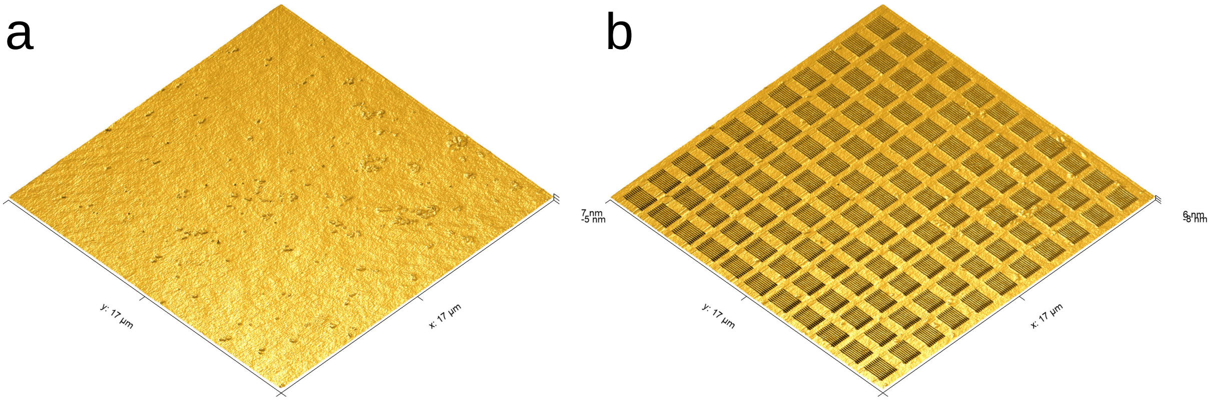

Figure S12: The AFM topology images in the fine step of the process method. **a** Before nano-lithography. **b** After nano-lithography.

In the fine step of the process method, the Setpoint value ranges from 3.0 to 4.0nA at an interval of 0.1 nA, while the scanning speed ranges from 5 to 55 µm/s at an interval of 5 µm/s. Fig. S12 illustrates the AFM topology image obtained during the fine step of the process method. Fig. S12a presents the topography of the sample surface after being spin-coated with PMMA prior to nanolithography. Following nanolithography and in-situ characterization, the nano-structures are obtained, as depicted in Fig.S12b. In the fine step of the process method, the gradient variation of the critical dimension and depth decrease. By utilizing this process method, the process parameters associated with the desired nano-structure can be attained.

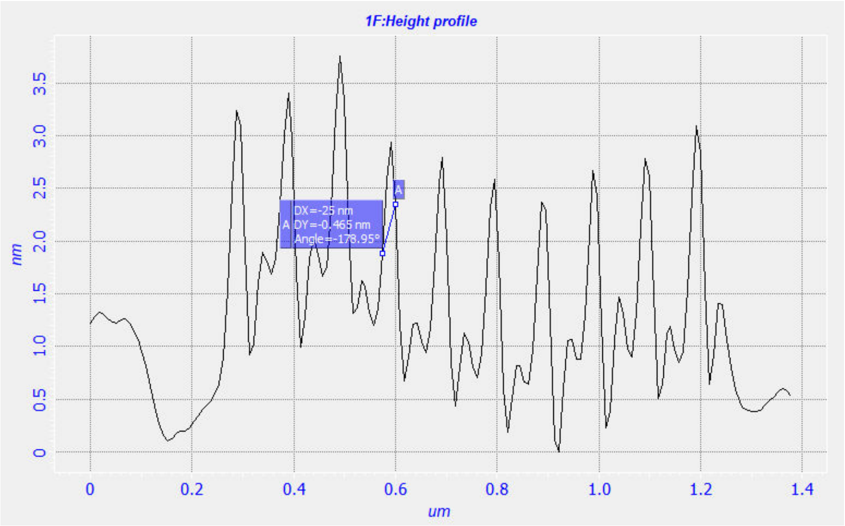

Figure S13: AFM probe in the SPL system.

Fig. S13 presents a cross-sectional view of the nano-lithography results for the parameter set with setpoint of 3.3 nA and speed of 25 µm/s. The FWHM of the structure with nano-lithography is approximately 25 nm.

Supplementary Note 8 Experimental results of b-SPL and o-SPL

To further verify the applicability of b-SPL and o-SPL to the proposed ML-based method, experiments on b-SPL and o-SPL are conducted. Fig. S14 shows the b-SPL experimental results with the proposed framework. The sample is a silicon wafer spin coated with PMMA. A bias voltage is applied between the sample and the probe, and the probe is connected to the negative pole. The SPL areas are successfully segmented with the proposed framework. And the global FWHM and depth are shown in Figs. S14d1-e4. With the above-mentioned global information, the process parameter optimization is accelerated.

Fig. S15 shows the b-SPL and m-SPL experimental results with the proposed framework. The sample is a silicon wafer spin coated with PMMA. A bias voltage is applied between the sample and the probe, and the probe is connected to the negative pole. The five vertical lines are fabricated using b-SPL, and the diagonal lines between the vertical lines are fabricated using m-SPL. The SPL areas are also successfully segmented with the proposed framework. The effectiveness of the proposed framework is experimentally verified for multiple SPLs. And the global FWHM and depth are shown in Figs. S15d1-e4. With the above-mentioned global information, the process parameter optimization is accelerated.

Fig. S16 shows the o-SPL experimental results with the proposed framework. The experimental conditions of o-SPL are similar to b-SPL, so we only conduct two sets of experiments for demonstration. The sample is a silicon wafer. A bias voltage is applied between the sample and the probe, and the probe is connected to the negative pole. Under the action of voltage, silicon is oxidized to silicon dioxide. Since the silicon dioxide protrudes on the surface of the sample, we choose the opposite value of the height for processing when performing grayscale processing. The other processes are not different from those of b-SPL. The SPL areas are also successfully segmented with the proposed framework. And the global FWHM and depth are shown in Figs. S16d1-e4. With the above-mentioned global information, the process parameter optimization is accelerated.

The above experiments show that the proposed ML-based framework is effective for m-SPL, o-SPL, and b-SPL.

Supplementary Note 9 Details of large-area nano-lithography

Furthermore, Fig. S17 showcases the SEM images of the QR code pattern with an overall size of 420×420 μm^2, consisting of 21×21 pixels. The nano-lithography pattern corresponding to each QR code pixel has a size of 20×20 μm^2. The pixels that form the QR code are processed in a sequential manner, with a nano-lithography process speed of 200 μm/s. Unlike other line-based structures, the uniformity of the nano-lithography areas in this pattern is critical. Therefore, we utilize continuous dense trajectories for nano-lithography. After nano-lithography, the PMMA material is plowed up.

Supplementary Note 10 Stitchless and stitched large-area SPL

To demonstrate the capability of stitch-free nano-fabrication with pitch of tens of nm and the overall scale of mm by the proposed SPL system, we conduct millimeter-area nano-lithography experiments based on the compliant nano-manipulator. The fabricated pattern encompasses an area measuring 1×1 mm^2 and consists of an array of parallel lines

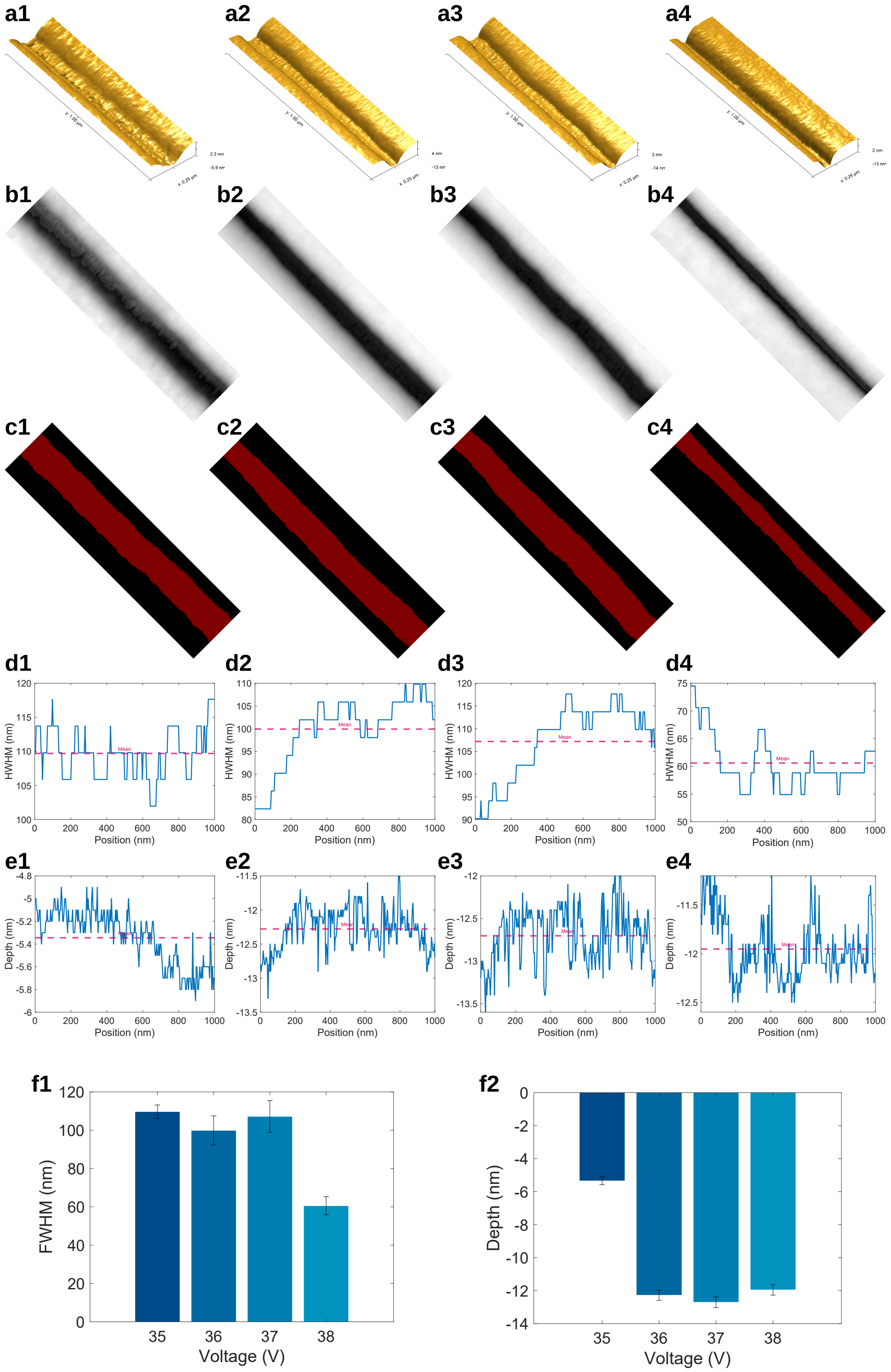

Figure S14: B-SPL experimental results with the proposed framework. **a1-a4** AFM images of b-SPL results at applied voltages of 35 V, 36 V, 37 V, and 38 V, respectively. **b1-b4** The grayscale images corresponding to **a1-a4**. **c1-c4** The SPL areas segmentation results corresponding to **a1-a4**. **d1-d4** The FWHM at each position corresponding to **a1-a4**. **e1-e4** The depth at each position corresponding to **a1-a4**. **f1** The relationship between FWHM and voltage. **f2** The relationship between depth and voltage.

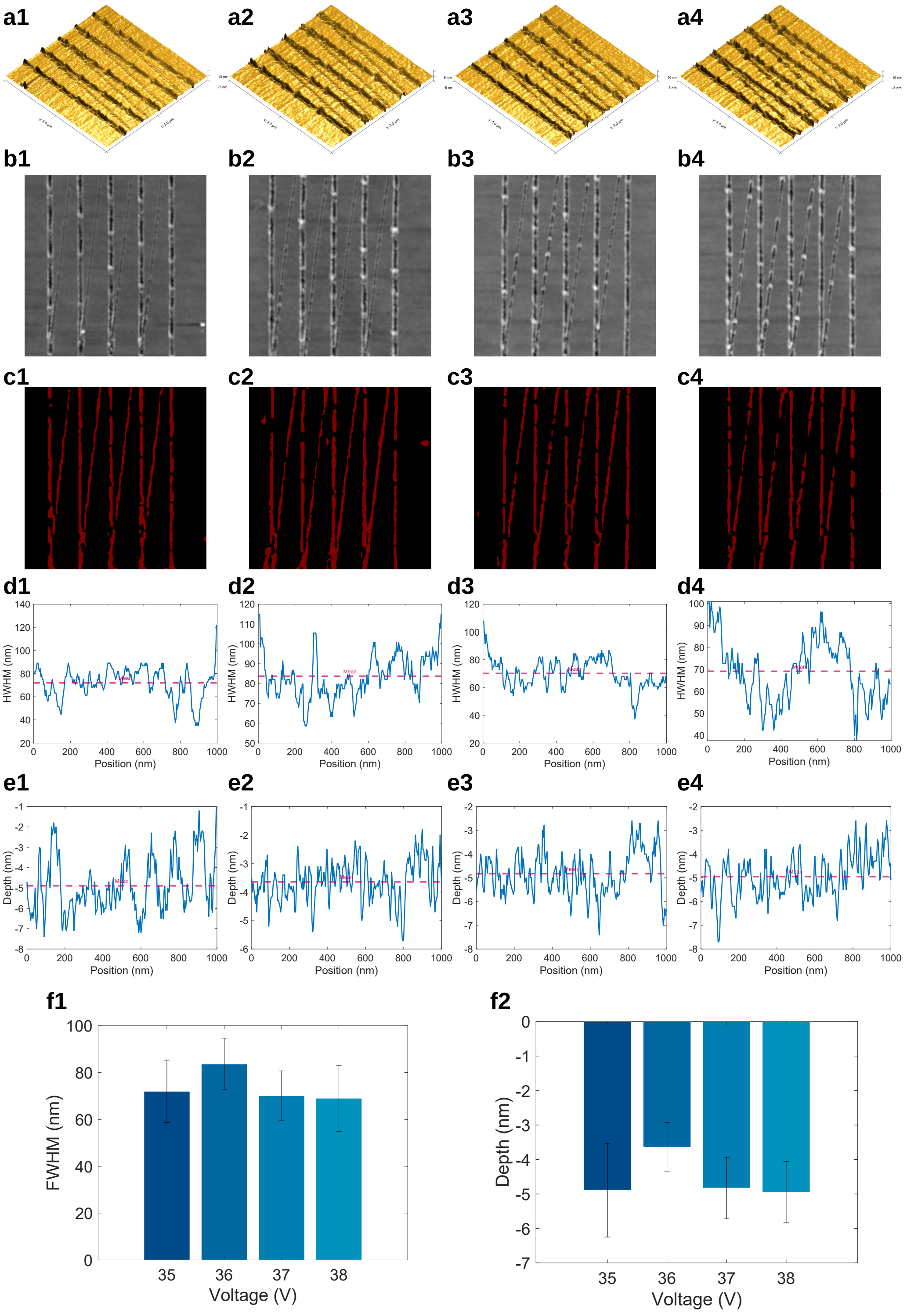

Figure S15: B-SPL and m-SPL experimental results with the proposed framework. **a1-a4** AFM images of b-SPL results at applied voltages of 35 V, 36 V, 37 V, and 38 V, respectively. **b1-b4** The grayscale images corresponding to **a1-a4**. **c1-c4** The SPL areas segmentation results corresponding to **a1-a4**. **d1-d4** The FWHM at each position corresponding to **a1-a4**. **e1-e4** The depth at each position corresponding to **a1-a4**. **f1** The relationship between FWHM and voltage. **f2** The relationship between depth and voltage.

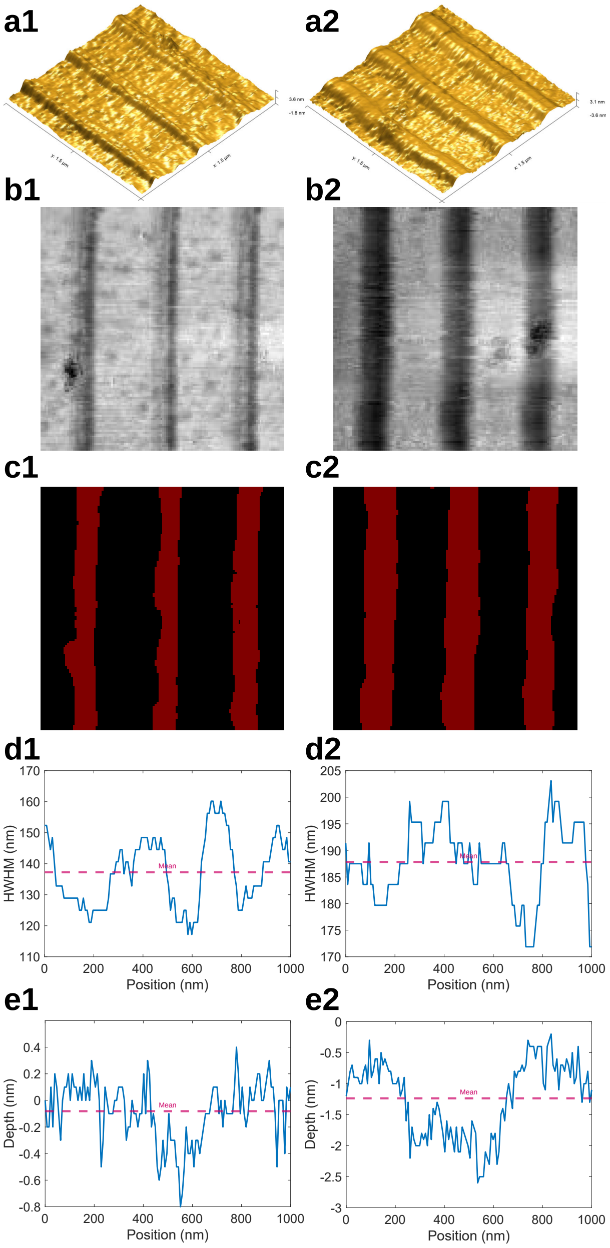

Figure S16: O-SPL experimental results with the proposed framework. **a1-a2** AFM images of b-SPL results at applied voltages of 50 V and 60 V, respectively. **b1-b2** The grayscale images corresponding to **a1-a2**. **c1-c2** The SPL areas segmentation results corresponding to **a1-a2**. **d1-d2** The FWHM at each position corresponding to **a1-a2**. **e1-e2** The depth at each position corresponding to **a1-a2**. **f1** The relationship between FWHM and voltage. **f2** The relationship between depth and voltage.

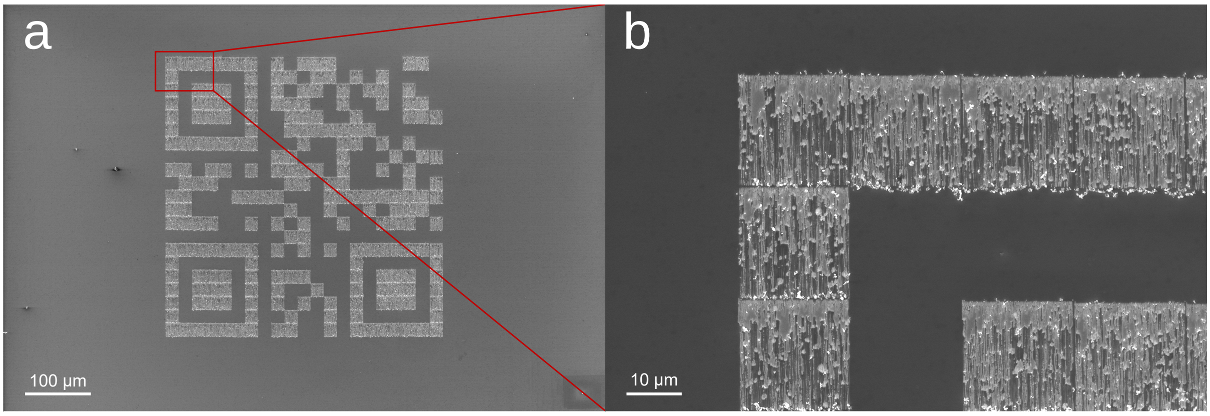

Figure S17: SEM images of the large-area nano-lithography. **a** SEM image of the large-area QR code (420×420 μm^2). **b** Partial enlarged view of **a**.

with a pitch of 500 mm. The proposed SPL system supports millimeter-level stroke, thus enabling direct processing of this pattern without stitching. However, conventional AFMs typically have a scanning range of less than 100×100 μm^2, and the existing approaches face challenges when directly applied to large-area fabrication and characterization. In order to showcase the SPL results in a stitched manner, the aforementioned large-area pattern is subdivided into 25 sub-patterns, each spanning an area of 200×200 μm^2. These sub-patterns are sequentially processed and subsequently stitched together to form a complete large-area pattern.

Fig. S18a and Fig. S18b show the SEM images of the large-area patterns. The speed of nanolithography is 1 mm/s. The stitchless method takes a total of 86 minutes and 43 seconds. And the stitched method takes a total of 166 minutes and 50 seconds. Compared with the stitched method, the stitchless nano-lithography result demonstrates an absence of stitching error, and the throughput is increased by 48%.

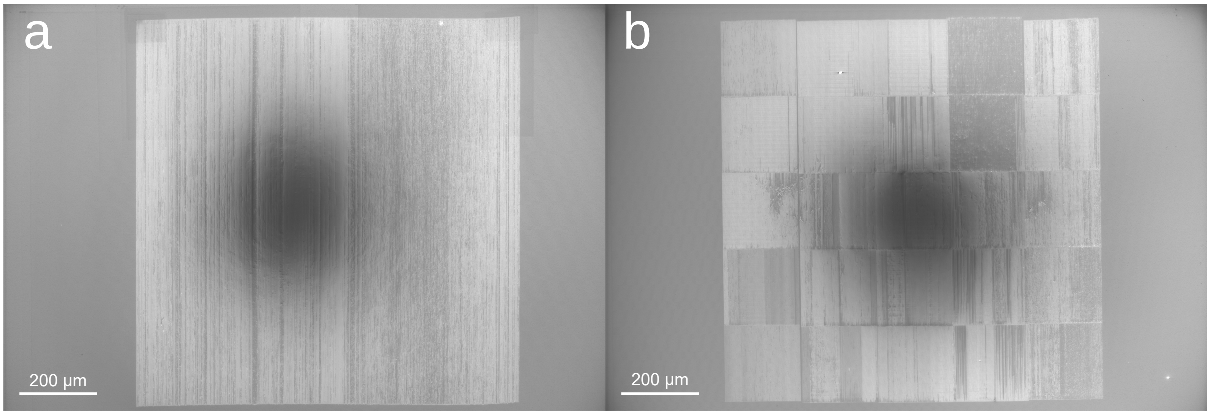

Figure S18: SEM images of the large-area patterns. **a** SEM image of the large-area pattern in a stitchless manner (1×1 mm^2). **b** SEM image of the large-area pattern in a stitched manner (1×1 mm^2).

Fig. S19 and Fig. S20 show the partial enlarged views of the upper left corner and the upper right corner of the large-area patterns, respectively. One can clearly see the SPL

results of different blocks of each sub-pattern in the stitched method.

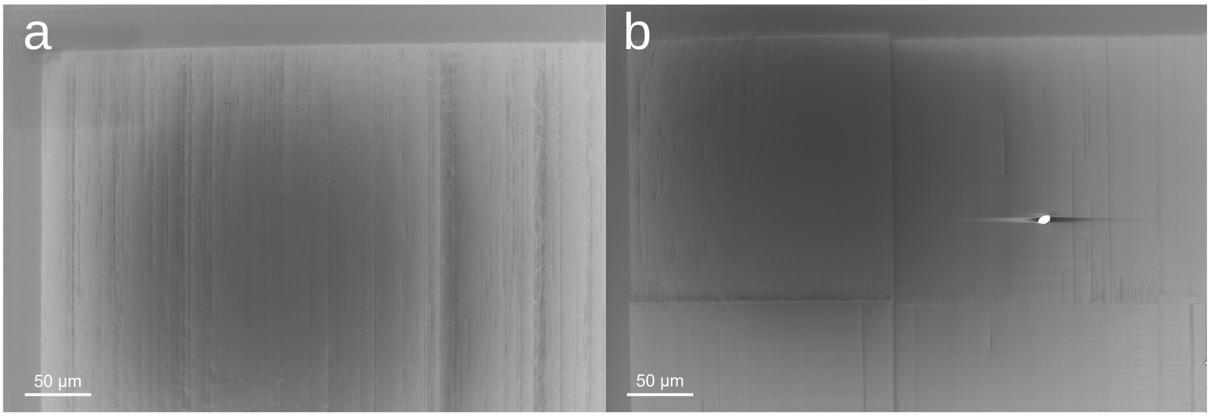

Figure S19: SEM images of the partial enlarged view of the upper left corner of the large-area patterns. **a** SEM image of the large-area pattern in a stitchless manner. **b** SEM image of the large-area pattern in a stitched manner.

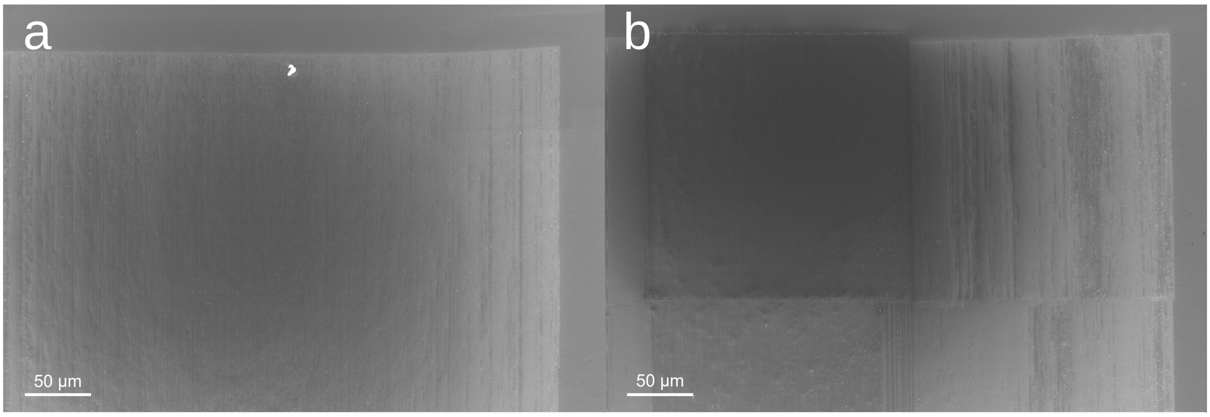

Figure S20: SEM images of the partial enlarged view of the upper right corner of the large-area patterns. **a** SEM image of the large-area pattern in a stitchless manner. **b** SEM image of the large-area pattern in a stitched manner.

Fig. S21 shows the SEM image of the junction of the four sub-patterns in a stitched manner. Among them, the four corners belong to four different sub-patterns. It can be seen that there are obvious stitching errors at the junction of each sub-pattern using the stitched manner.

Fig. S22a and Fig. S22b show the partial enlarged SEM images of the above two large-area patterns processed by different methods using the same probe. Following several hours of utilization, both patterns produced through SPL exhibit critical dimensions of approximately 20 nm. Experiments verify that SPM can support large-area and long-time etching, and the accuracy is almost unaffected.

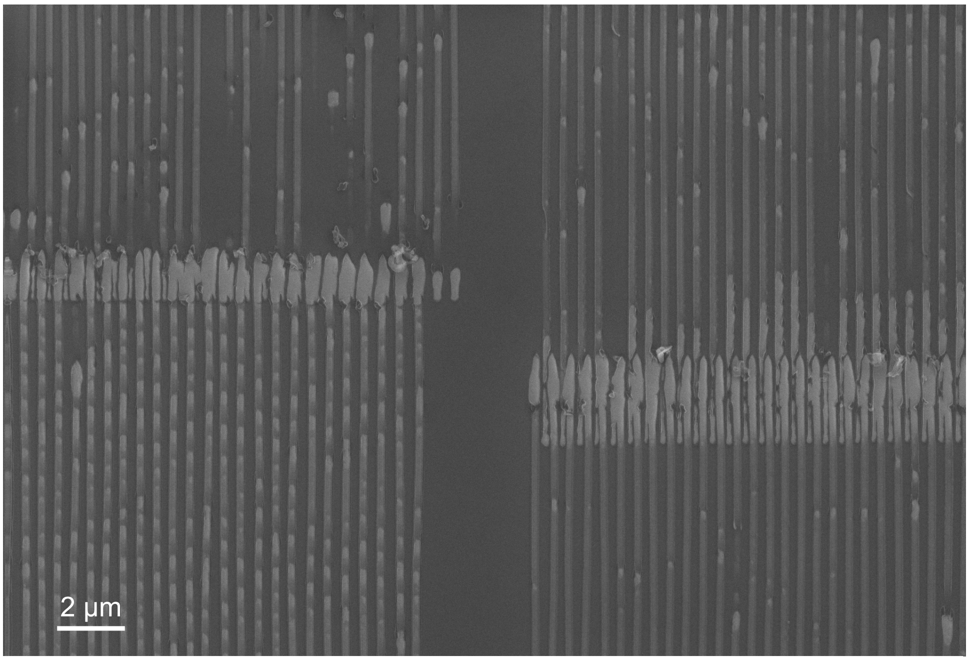

Figure S21: SEM image of the junction of the four sub-patterns in a stitched manner.

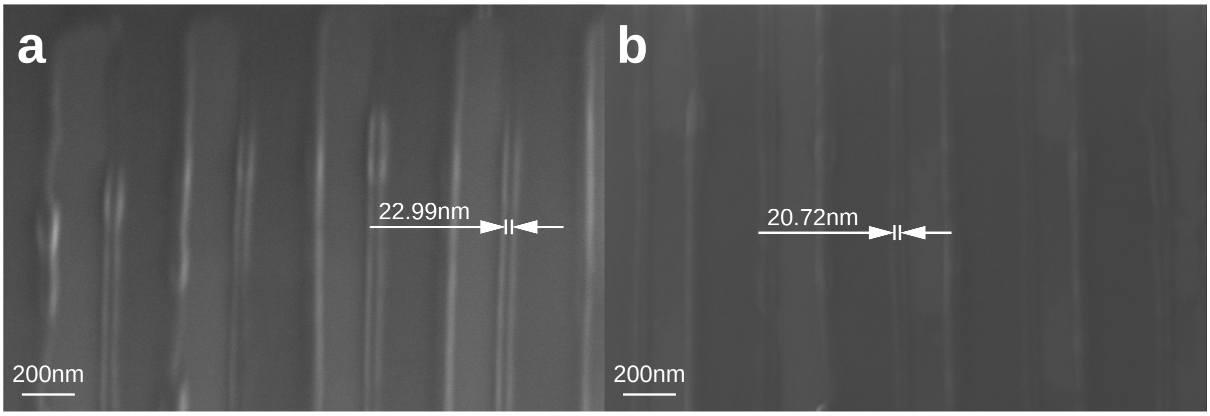

Figure S22: Partial enlarged views of the large-area patterns. **a** SEM image of the large-area pattern in a stitchless manner. **b** SEM image of the large-area pattern in a stitched manner.

Supplementary Note 11 SPL results on hard substrates

To further verify the accuracy of long-time SPL on hard substrates, we choose polished copper as the substrate. During the process, a tapping mode AFM probe with an aluminum reflective coating (Tap300DLC, BudgetSensors®, Sofia, Bulgaria) is used for nanolithography. These probes have long cantilevers with diamond-like-carbon coating on tip side of the cantilever that are 15 µm high at the apex, and a typical curvature radius of approximately 15 nm. The nominal spring constant k is 40 N/m, and the resonant frequency is 300 kHz. The lithography speed is 0.3 µm/s.

Fig. S23 shows the SPL results on copper substrate. The pattern is an array of parallel lines with a pitch of 500 nm. Fig. S24 shows the height information of A-A cross-section line in Fig. S23. The critical dimension of the lines is 91.9 nm.

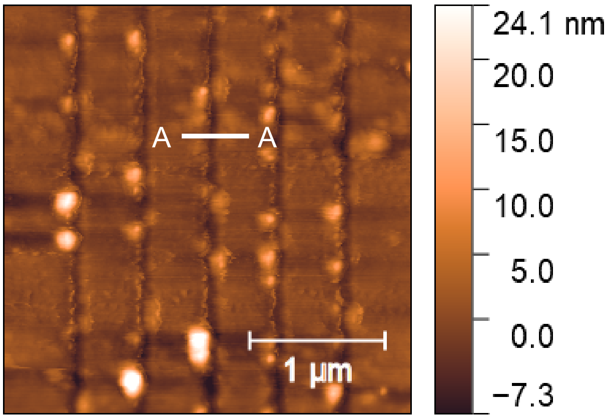

Figure S23: SPL results on copper substrate.

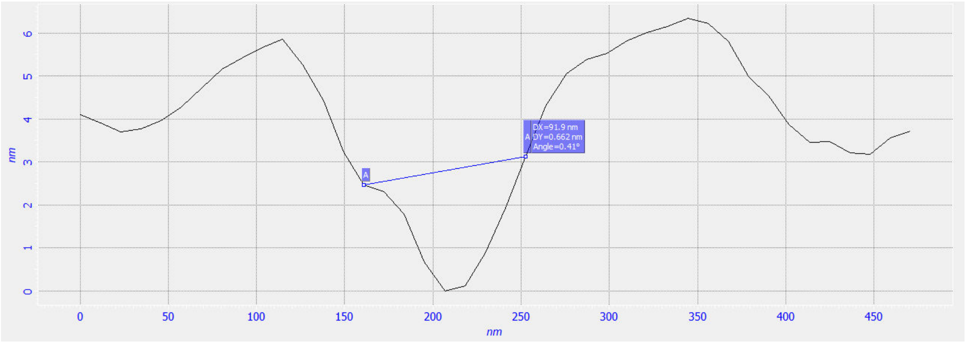

Figure S24: Height information of A-A cross-section line in Fig. S23.

To further verify the capability of long-time SPL on hard substrates, we perform continuous SPL on copper substrates for more than 3 hours. Fig. S25 shows a part of the SPL result.

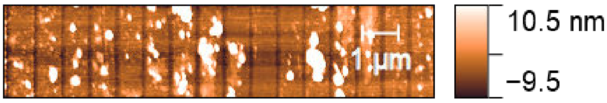

Figure S25: Long-time SPL results on copper substrate.

Fig. S26 shows the final lines after the long-time SPL. And Fig. S27 shows the height information of A-A cross-section line in Fig. S26. The critical dimension of the lines is

89.2 nm, which is consistent with the result before long-time SPL. Experiments verify that SPM can support long-time SPL on hard substrates, and the accuracy is hardly affected.

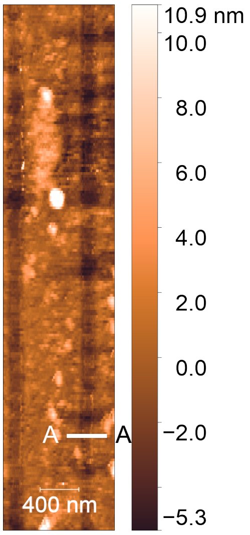

Figure S26: Long-time SPL results on copper substrate.

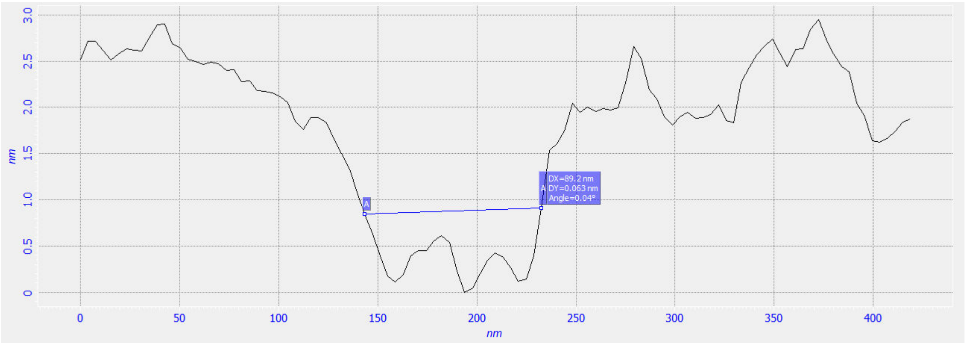

Figure S27: Height information of A-A cross-section line in Fig. S26.

To study the effect of substrate hardness on SPL results, we further choose polished silicon wafers as substrates. The Tap300DLC probe is chosen, which is the same as the previous probe in the experiments on copper substrates. The lithography speed is 0.3 µm/s. Fig. S28 shows the SPL results on silicon substrate. The pattern is an array of parallel lines with a pitch of 500 nm. Fig. S29 shows the height information of A-A cross-section line in Fig. S28. The critical dimension of the lines is 109.6 nm. And the depth is 1.107 nm. The aspect ratio is calculated to be 0.010. The experimental aspect ratio of the silicon substrate is only 27.8% of that of the copper substrate (0.036). Compared with the soft material (PMMA, aspect ratio: 0.373), as shown in Figs. S30-S31, the harder substrate, the smaller aspect ratio.

Supplementary Note 12 Scanning probe in the SPL system

A tapping mode AFM probe with aluminum reflective coating (Tap190Al-G, BudgetSensors®, Sofia, Bulgaria) is selected for ultra-large scale characterization, as shown in Fig. S32.

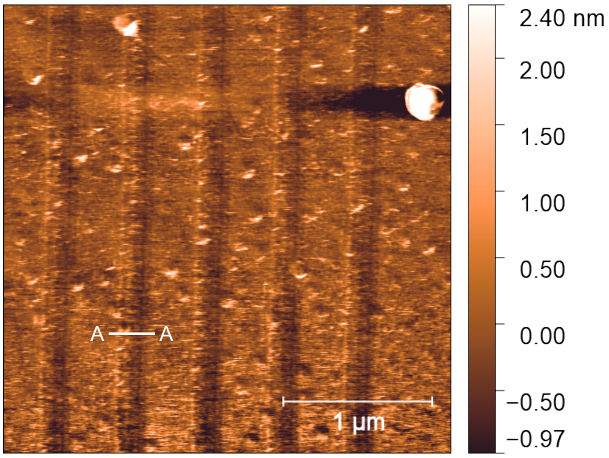

Figure S28: SPL results on silicon substrate.

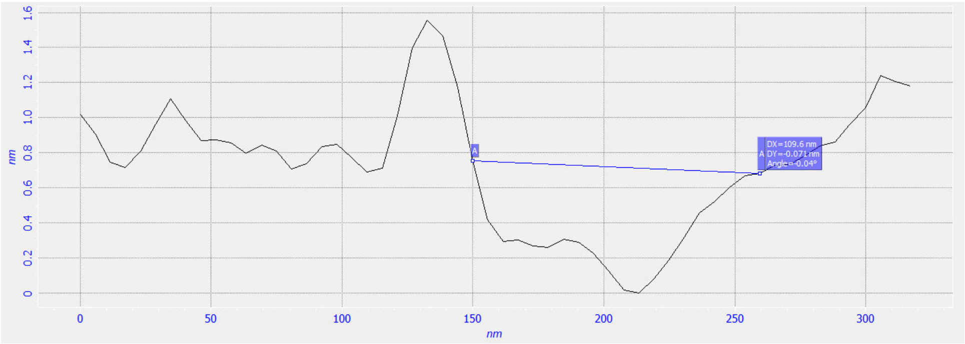

Figure S29: Height information of A-A cross-section line in Fig. S28.

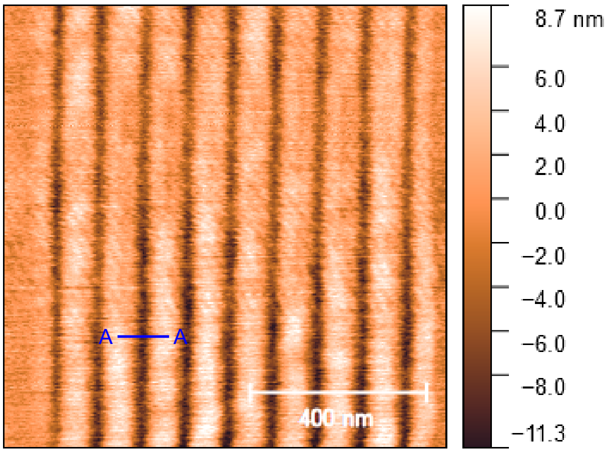

Figure S30: SPL results on PMMA substrate.

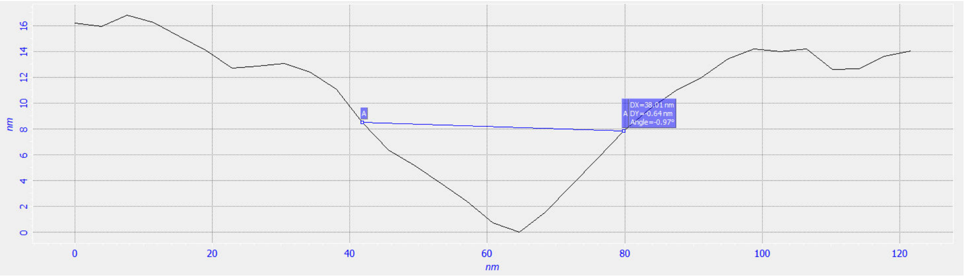

Figure S31: Height information of A-A cross-section line in Fig. S30.

Tap190Al-G probes are long cantilevers, with conical tips 15 µm high at the apex. The typical curvature radius is approximately 10 nm. The nominal spring constant k is 48 N/m, and the resonant frequency is 190 kHz. The free amplitude of the probe during the tapping mode is about 12 nm. The experiments are performed in the ambient environment (room temperature of about 25°C and relative humidity around 50%).

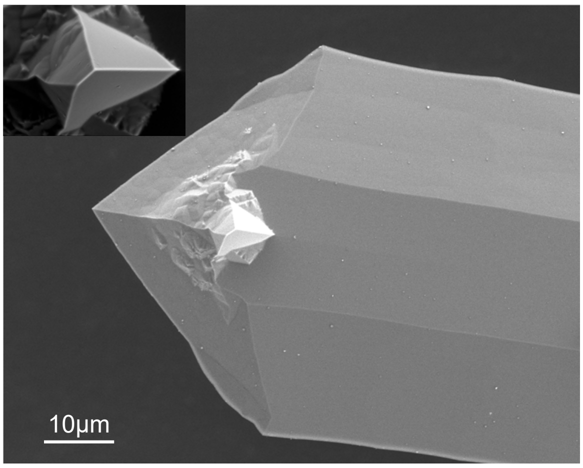

Figure S32: AFM probe in the SPL system.

Supplementary Note 13 Comparisons of different nano-lithography methods

We compare the SPL results with other lithography methods, as shown in Tab. S3 [S8]. Note that the processing results (resolution and speed) are not standard, we list typical data of different nano-lithography methods in Tab. S3. It is evident that SPL exhibits substantially higher throughput compared to EBL and FIB. Furthermore, SPL achieves a smaller critical dimension than common photolithography. Additionally, SPL offers advantages in terms of environmental conditions and cost considerations.

References

[S1] Zhen Zhang, Xiaodong Yang, and Peng Yan. Large dynamic range tracking of an XY compliant nanomanipulator with cross-axis coupling reduction. *Mechanical Systems and Signal Processing*, 117:757–770, 2019.

[S2] Shorya Awtar and Gaurav Parmar. Design of a large range XY nanopositioning system. *Journal of Mechanisms and Robotics*, 5(2):021008, 2013.

Table S3: Comparisons of different nano-lithography methods.

	EBL	FIB	Common photolithography	SPL (this work)
Resolution	5 nm [S9]	5 nm [S10]	$0.6 \sim 5$ μm [S11]	~ 20 nm
Speed	40 nm/min [S12], and 58 nm/min [S13] against different resists	190 nm/s [S14], and 0.05 μm^3/s [S15] against different resists	17 mm^2/min (0.6 μm minimum feature size), 50 mm^2/min (1 μm minimum feature size), 120 mm^2/min (2 μm minimum feature size), and 180 mm^2/min (5 μm minimum feature size) [S16]	1 mm/s
Environment	Vacuum	Vacuum	Vacuum or ambient	Ambient
Cost	High	High	High	Low

[S3] Ammar Al-Jodah, Bijan Shirinzadeh, Mohammadali Ghafarian, Tilok Kumar Das, Yanling Tian, Dawei Zhang, and Fujun Wang. Development and control of a large range XYθ micropositioning stage. *Mechatronics*, 66:102343, 2020.

[S4] Dejun Guo, William S Nagel, Garrett M Clayton, and Kam K Leang. Spatial-temporal trajectory redesign for dual-stage nanopositioning systems with application in AFM. *IEEE/ASME Transactions on Mechatronics*, 25(2):558–569, 2020.

[S5] Andrew J Fleming and Yuen Kuan Yong. An ultrathin monolithic XY nanopositioning stage constructed from a single sheet of piezoelectric material. *IEEE/ASME Transactions on Mechatronics*, 22(6):2611–2618, 2017.

[S6] Mingxiang Ling, Junyi Cao, Zhou Jiang, Minghua Zeng, and Qisheng Li. Optimal design of a piezo-actuated 2-DOF millimeter-range monolithic flexure mechanism with a pseudo-static model. *Mechanical Systems and Signal Processing*, 115:120–131, 2019.

[S7] Yoshihiko Koseki, Tamio Tanikawa, Noriho Koyachi, and Tatsuo Arai. Kinematic analysis of a translational 3-DOF micro-parallel mechanism using the matrix method. *Advanced Robotics*, 16(3):251–264, 2002.

[S8] Pengfei Fan, Jian Gao, Hui Mao, Yanquan Geng, Yongda Yan, Yuzhang Wang, et al. Scanning probe lithography: state-of-the-art and future perspectives. *Micromachines*, 13(2):228, 2022.

[S9] Anushka S Gangnaik, Yordan M Georgiev, Gillian Collins, and Justin D Holmes. Novel germanium surface modification for sub-10 nm patterning with electron beam lithography and hydrogen silsesquioxane resist. *Journal of Vacuum Science & Technology B*, 34(4):041603, 2016.

[S10] Nitul S Rajput and Xichun Luo. FIB Micro-/Nano-fabrication. 2015.

[S11] Víctor J Gómez, Mariusz Graczyk, Reza Jafari Jam, Sebastian Lehmann, and Ivan Maximov. Wafer-scale nanofabrication of sub-100 nm arrays by deep-UV displacement talbot lithography. *Nanotechnology*, 31(29):295301, 2020.

[S12] Jae Beom Yoo, Sang-Wook Park, Ha Na Kang, Hemant S Mondkar, Kyunghwa Sohn, Hyun-Mi Kim, Ki-Bum Kim, and Haiwon Lee. Triphenylsulfonium salt methacrylate bound polymer resist for electron beam lithography. *Polymer*, 55(16):3599–3604, 2014.

[S13] DX Yang, Andreas Frommhold, X Xue, RE Palmer, and APG Robinson. Chemically amplified phenolic fullerene electron beam resist. *Journal of Materials Chemistry C*, 2(8):1505–1512, 2014.

[S14] Ampere A Tseng. Recent developments in nanofabrication using focused ion beams. *Small*, 1(10):924–939, 2005.

[S15] Jiwang Yan and Nozomi Takayama. *Micro and nanoscale laser processing of hard brittle materials*. Elsevier, 2019.

[S16] Durham Magneto Optics Ltd. MicroWriter ML3 Pro: a direct-write photolithography machine for rapid prototyping in R&D laboratories and small clean rooms. [EB/OL]. `https://www.durhammagnetooptics.com/?product=microwriter` Accessed July 8, 2023.
